# Advances in gene and cellular therapeutic approaches for Huntington’s disease

**DOI:** 10.1093/procel/pwae042

**Published:** 2024-08-09

**Authors:** Xuejiao Piao, Dan Li, Hui Liu, Qing Guo, Yang Yu

**Affiliations:** Clinical Stem Cell Research Center, Peking University Third Hospital, Beijing 100191, China; Clinical Stem Cell Research Center, Peking University Third Hospital, Beijing 100191, China; Clinical Stem Cell Research Center, Peking University Third Hospital, Beijing 100191, China; Clinical Stem Cell Research Center, Peking University Third Hospital, Beijing 100191, China; Clinical Stem Cell Research Center, Peking University Third Hospital, Beijing 100191, China; Beijing Key Laboratory of Reproductive Endocrinology and Assisted Reproductive Technology and Key Laboratory of Assisted Reproduction, Ministry of Education, Center of Reproductive Medicine, Department of Obstetrics and Gynecology, Peking University Third Hospital, Beijing 100191, China

**Keywords:** Huntington’s disease (HD), Huntingtin gene (*HTT*), gene therapy, cell therapy, interdisciplinary research

## Abstract

Huntington’s disease (HD) is an inherited neurodegenerative disorder caused by the abnormal expansion of CAG trinucleotide repeats in the Huntingtin gene (*HTT*) located on chromosome 4. It is transmitted in an autosomal dominant manner and is characterized by motor dysfunction, cognitive decline, and emotional disturbances. To date, there are no curative treatments for HD have been developed; current therapeutic approaches focus on symptom relief and comprehensive care through coordinated pharmacological and nonpharmacological methods to manage the diverse phenotypes of the disease. International clinical guidelines for the treatment of HD are continually being revised in an effort to enhance care within a multidisciplinary framework. Additionally, innovative gene and cell therapy strategies are being actively researched and developed to address the complexities of the disorder and improve treatment outcomes. This review endeavours to elucidate the current and emerging gene and cell therapy strategies for HD, offering a detailed insight into the complexities of the disorder and looking forward to future treatment paradigms. Considering the complexity of the underlying mechanisms driving HD, a synergistic treatment strategy that integrates various factors—such as distinct cell types, epigenetic patterns, genetic components, and methods to improve the cerebral microenvironment—may significantly enhance therapeutic outcomes. In the future, we eagerly anticipate ongoing innovations in interdisciplinary research that will bring profound advancements and refinements in the treatment of HD.

## Introduction

Huntington’s disease (HD) is an inherited neurodegenerative condition that inevitably progresses towards a fatal outcome. Caused by an abnormal expansion of CAG triplet repeats in the Huntingtin gene (*HTT*), located at the 16.3 position of the short arm of chromosome 4, it acts as an autosomal dominant affliction. Globally, HD manifests at a rate of 2.71 per 100,000 people, but this frequency increases to between 10.6 and 13.7 per 100,000 people in western nations, including Europe, the USA, and Australia (Huntington disease, 2015; Evans et al., 2013; Fisher and Hayden, 2014; Pringsheim et al., 2012). HD was meticulously described by Dr. George Huntington in 1872 and bears his name (Bhattacharyya, 2016). It presents with a triad of symptoms: disturbances in voluntary movement, cognitive decline, and emotional disturbances (Martin and Gusella, 1986). While its onset can span from the teenage years to later adulthood, the age of onset is influenced by various factors, including the individual’s CAG repeat load and environmental influences, with most individuals displaying symptoms in approximately their mid-forties. HD tends to relentlessly progress over a course of 15–20 years post onset. A common and fatal complication is aspiration pneumonia, which often leads to mortality (Pringsheim et al., 2012; Shiwach and Norbury, 1994).

Typically, the majority of healthy individuals have a CAG repeat sequence within the *HTT* gene that ranges from 9 to 35 units. In contrast, patients with HD often exhibit more than 35 CAG repeats, and the number of CAG repeats is directly correlated with an earlier onset and increased severity of the disease. A repeat count greater than 55 frequently results in disease manifestation during adolescence. Research indicates that there is an inverse relationship between the number of pathogenic, abnormally expanded CAG repeats and the age of onset of HD; moreover, there is a direct correlation between repeat length and the severity of the condition under the same disease duration. The length of the CAG repeat sequence is a significant factor in the clinical presentation of the disease (Stine et al., 1993).

Additionally, such as other repeat expansion diseases, a prominent feature of HD is the high instability of these repeat sequences. Both intergenerationally and somatically, these sequences progressively expand over time in a cell- or tissue-specific manner. Notably, in HD, the central nervous system (CNS), which is most severely affected by the *HTT* mutation, exhibit the most pronounced CAG expansion (Mouro Pinto et al., 2020). These observations, combined with the increasing genetic evidence from genome-wide association studies of HD patients (Genetic Modifiers of Huntington’s Disease (GeM-HD), 2019), support the hypothesis that the gradual increase in repeat length within somatic tissues contributes to the pathogenesis. As a result, the progression of HD can be understood as comprising two key components: the most important compartment somatic CAG expansions, which act as a rate-determining mechanism influencing the timing of disease onset, and another is a toxicity mechanism, which initiates cellular damage when the CAG repeat expansion exceeds a critical threshold (Aldous et al., 2024; Belgrad and Khvorova, 2024; Hong et al., 2021).However, it is important to note that once HD patients manifest symptoms, the duration of the disease is not related to the length of the CAG repeats and is determined by factors other than the initial trigger of the disease mechanism (Finkbeiner, 2011). Beyond expanded CAG repeats, epigenetic regulation and post-translational modifications also have a significant impact on the disease’s occurrence (Genetic Modifiers of Huntington's Disease (GeM-HD) Consortium, 2015; Francelle et al., 2017).

## *HTT* gene biological functions

The *HTT* gene spans approximately 203 kb, consists of 67 exons, and encodes a protein with a molecular weight of approximately 348 kDa. The gene is expressed universally in the body and plays a pivotal role in human development. It has two distinct transcripts that vary in their relative presence in fetal versus adult tissues. The most substantial transcript, approximately 13.7 kb, has been primarily found in the CNS of both adults and fetuses, with a high concentration in the cerebellum. In contrast, the smaller transcript, approximately 10.3 kb, is expressed in a wide range of bodily tissues. The differing expression patterns suggest that each transcript variant may serve unique functions in normal physiological operations (Guo et al., 2018). As a scaffold protein, the HTT protein is instrumental in regulating numerous proteins and cellular functions due to its intricate structure and various interaction points (Zuccato et al., 2010). It is implicated in a myriad of functions, such as modulating transcription, governing axonal transport, managing endosome and organelle trafficking, facilitating vesicle recycling, coordinating cell division, and overseeing ciliogenesis (Saudou and Humbert, 2016).

HTT serves as a regulator of gene transcription, collaborating with an array of transcription factors and regulators to amplify or curtail gene expression (Dunah et al., 2002; Marcora et al., 2003; Steffan et al., 2000). Through its interactions with transcription activators (Holbert et al., 2001) and repressors (Yohrling et al., 2003; Zuccato et al., 2003), HTT can enhance the function of transcription factors while tempering the activity of inhibitors, thereby modulating gene expression. HTT also engages with nuclear receptors, playing a role in regulating transcriptional mechanisms (Futter et al., 2009). Within the nucleus, HTT may act as a scaffold for transcriptional complexes and participate in regulating chromatin remodeling (Dunah et al., 2002). Additionally, the role of HTT in intracellular dynamics may have implications for transcription.

As pivotal orchestrators in the transport of intracellular organelles, HTT engages in direct interactions with motor proteins or indirectly liaises through associated adaptor proteins. It transports a diverse array of organelles, including presynaptic vesicles (Zala et al., 2013), brain-derived neurotrophic factor (BDNF)-infused vesicles (Gauthier et al., 2004), autophagosomes (Wong and Holzbaur, 2014), endosomes and lysosomes (Caviston et al., 2011), thus increasing transport efficacy. Additionally, HTT enhances vesicular trafficking by binding to glyceraldehyde 3-phosphate dehydrogenase （GAPDH） present on vesicles and collaborating with molecular motors to harmonize energy generation with expenditure, ensuring seamless transport. By undergoing phosphorylation at the S421 residue, HTT not only accelerates vesicle velocity but also provides precise directional control (Colin et al., 2008).

The HTT protein plays a vital role in the cellular division process, engaging in the positioning of spindle poles and regulating the activity of transport proteins. Loss of HTT function during mitosis leads to erroneous spindle positioning (Elias et al., 2014). The function of HTT in spindle orientation is evolutionarily conserved; spindle placement errors can be triggered in Drosophila neural progenitor cells (NPCs) by silencing HTT. Conversely, the expression of Drosophila HTT can rectify spindle orientation defects in mammalian cells caused by *HTT* depletion (Godin et al., 2010).

Moreover, HTT is indispensable for ciliogenesis, occupying a prominent position at the base of primary neuronal cilia, in photoreceptive rods and cones in the retina, and in multiciliated cells. It mediates the delivery of essential proteins to the pericentriolar material via interactions with proteins such as HAP1 and PCM1. A deficiency in HTT disrupts the retrograde transport of PCM1 and interferes with ciliogenesis, resulting in a reduced number of cilia (Keryer et al., 2011). The phosphorylation of the N-terminal domain of HTT, in conjunction with its interactions with HIP1, is vital for the targeted localization of the protein and its governing role within the cellular context (Badano et al., 2005).

## HD pathogenesis

The expansion of CAG trinucleotide repeats within the *HTT* gene is a key factor in causing neuronal dysfunction and death. The formation of HD is largely due to the toxic gain-of-function mechanism of the mutant Huntingtin (mHTT) protein. This expansion caused by the repeat sequences leads to the translation of polyglutamine (polyQ) stretches at the N-terminus of the HTT protein, resulting in the formation of both the toxic full-length mHTT protein and exon 1 N-terminal fragments. The abnormal polyQ stretch induces structural alterations in the protein, leading to abnormal protein aggregation that cannot be cleared by cellular systems such as lysosomes and the ubiquitin-proteasome system. These aggregates accumulate in both the cytoplasm and nucleus, forming deposits that aberrantly interact with other proteins and disrupt their normal functions. This disruption adversely affects cellular proteostasis, nuclear pore complex functionality, axonal transport, transcription, translation, mitochondrial function, and synaptic signalling, ultimately resulting in neuronal damage (Jones and Hughes, 2011; Ross and Tabrizi, 2011).

Although polyQ expansion is typically associated with disease pathogenesis through a mechanism of gain-of-toxicity, it has also been shown to trigger a wide spectrum of other neurodegenerative disorders. These include eight subtypes of spinocerebellar ataxia (SCA), SCA1, SCA2, SCA3, SCA6, SCA7, SCA12, and SCA17, and a novel SCA subtype caused by intragenic CAG repeat expansion in THAP11, in addition to dentatorubral-pallidoluysian atrophy (DRPLA), Huntington disease-like 2, and spinal and bulbar muscular atrophy (Robitaille et al., 1997; Stoyas and La Spada, 2018; Tan et al., 2023). The associated conditions are linked to aberrant expansions of polyQ, with enlargement of different proteins causing targeted neuronal damage. This, in turn, results in the malfunction of distinct brain regions. The pathology specific to these diseases arises from proteins harboring expanded polyQ mutations. Hence, while the pivotal pathological factor is the toxic gain-of-function induced by polyQ expansions, loss-of-function mutations in the *HTT* gene contribute to the onset and progression of HD.

Pathologically altered HTT protein has a selective neurodegenerative impact on certain neurons. In the case of HD, neuronal cell death preferentially occurs within the striatum or the basal ganglia and subsequently strongly affects layers III–V of the cerebral cortex, leaving the cerebellum largely untouched. As the disease progresses, other brain areas, including the hippocampus and hypothalamus, may also sustain damage (Duyao et al., 1993). Notably, in the striatum of HD patients, medium spiny neurons (MSNs)—which constitute 95% of striatal neurons and function as GABAergic neurons—are the most prominently affected. These neurons form dendritic spines that receive projections from cortical neurons, while their efferent outputs are principally linked to the internal and external segments of the globus pallidus and the reticular part of the substantia nigra. The decline in striatal volume along with cortical atrophy commences nearly a decade prior to symptomatic manifestation (Aylward et al., 2011; Douaud et al., 2006; Tabrizi et al., 2012).

Alterations in the striatum and cortical regions are intimately linked to dysfunction of the *HTT* gene. Intriguingly, the striatum is nearly wholly dependent on cortical input for BDNF, as it cannot produce BDNF itself (Finkbeiner, 2011). The HTT protein plays crucial roles in the transcription and transport of BDNF, aiding in its transcription and facilitating synaptic transfer from the cortex to the striatum (Gauthier et al., 2004; Zuccato et al., 2001). When BDNF is released in corticostriatal synapses, HTT controls retrograde transport of the BDNF–TrkB receptor, which is mediated by the interaction of HTT with the dynein motor protein complex IC-1B (Liot et al., 2013). Given the critical dependency of striatal neurons on BDNF signals generated and conveyed from the cortex, the deterioration of these neurons observed in HD patients is understandable. Mutations in *HTT* impede the protein’s normal functions, thereby disrupting BDNF synthesis and transport and precipitating the degeneration of striatal neurons. While changes in the CNS are the most prominent clinical feature of HD, patients also suffer from metabolic and immune dysregulation, muscular atrophy, weight reduction, cardiac failure, testicular atrophy, and osteoporosis (van der Burg et al., 2009).

Currently, there is no definitive cure for HD, and therapeutic efforts are aimed squarely at managing the diverse symptoms of HD through a combination of pharmacological and non-pharmacological interventions. Tetrabenazine, Austedo (deutetrabenazine), and Ingrezza (valbenazine capsules) are major treatments for alleviating chorea. Additionally, the use of antidepressants and antipsychotics such as Haloperidol, Sulpiride, and Quetiapine is recommended to improve chorea and psychiatric abnormalities (Coppen and Roos, 2017; Furr Stimming et al., 2023). Moreover, several preclinical drugs are under development. Research has found that Curcumin can ameliorate HD-related immune (Dhankhar et al., 2023) and metabolic (Aditi et al., 2022) dysregulation in *Drosophila* Model, thus offering some therapeutic benefits for HD. Additionally, studies have indicated that ganglioside GM1 can induce the phosphorylation of mHTT protein, restoring motor function and psychiatric disorders in HD mice, showcasing a broad range of beneficial effects (Alpaugh et al., 2017; Di Pardo et al., 2012). Despite significant refinements in HD disease management over the past two decades, there is still an absence of groundbreaking progress that could genuinely eradicate HD. Personalized HD management necessitates careful consideration of symptom variations, meticulous management of adverse drug reactions, potential complications, and drug interactions (Ferguson et al., 2022; Frank, 2014). Nonetheless, it is encouraging to note that numerous novel therapies aimed at targeting the root causes of this disease and reducing the levels of mHTT are being explored and developed.

The journey of medical discovery is a relentless pursuit of advancement. This review endeavours to illuminate the landscape of existing and emerging gene and cell therapeutic strategies for HD, aiming to provide a comprehensive overview that deciphers the complex nature of this disorder. At the same time, we provide a forward-looking perspective on treatment strategies for HD. Given the complexity of HD, a multifaceted treatment approach that addresses various aspects, including different cell types, epigenetic modifications, genetic factors, and the enhancement of the brain’s microenvironment, is likely to significantly enhance treatment outcomes. We anticipate that ceaseless innovation in interdisciplinary research will lead to significant improvements and refinements in HD treatments.

## Gene therapy for HD

**Figure 1.**
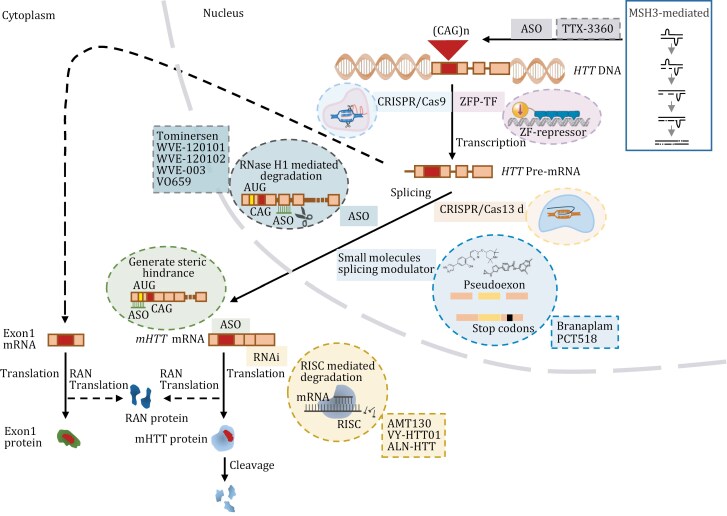
Schematic of targeted gene therapy strategies for Huntington’s disease across various phases of mHTT protein synthesis. The red highlights within the DNA, RNA, and protein models denote the toxic CAG repeat expansion and its consequent polyglutamine stretch (polyQ). The inset in the upper-right corner delineates the mechanism governing the expansion of CAG repeats. Therapeutic strategies are differentiated by a colour-coded array of boxes, with each shade representing a unique method of intervention. Oval outlines specify the underlying mechanisms driving the different treatments, while rectangular outlines correspond to the actual therapeutic agents in use. The dotted arrows throughout the figure suggest a hypothesized process for the generation of alternative toxic variants.

**Figure 2.**
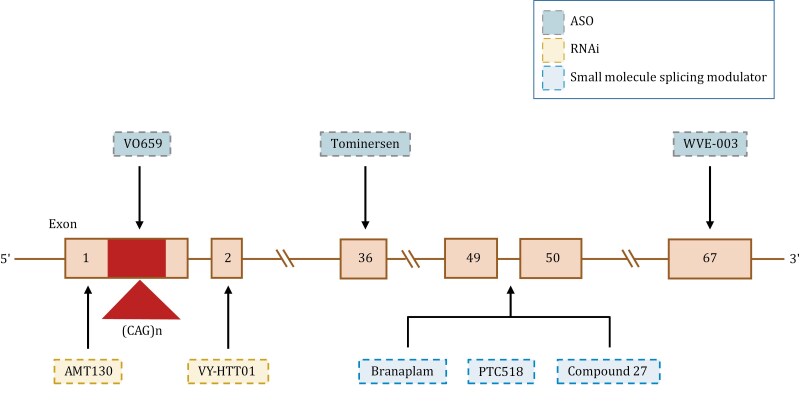
Overview of therapeutic targets of gene therapeutic drugs. Illustrates that therapeutic interventions are specifically designed to modulate the *mHTT* gene at various points along its sequence, aiming to reduce the production of mHTT. ASOs such as VO659, Tominersen, and WVE-003 were designed to bind specifically to the pre-mRNA of exon 1, exon 36, and exon 67, respectively, leading to mRNA degradation. RNAi therapies, including drugs like AMT130 and VY-HTT01, target exon 1 and exon 2, respectively, triggering the degradation of *HTT* mRNA and thereby reducing protein synthesis. Small molecule splicing modulator drugs such as Branaplam, PCT518, and Compound 27 can lower HTT levels by enhancing pseudoexon inclusion between exon 49 and exon 50 of the *HTT* gene.

Advances in gene therapy for the treatment of HD are progressing rapidly. The disease is caused by a mutation in a single gene; therefore, the primary goal of treatment is to minimize the impact of an expansion of CAG repeats at the source of the m*HTT* gene (Tabrizi et al., 2019a). To achieve this goal, researchers are working on two fronts. First, they are seeking to correct or delete the mutated HTT gene at the DNA level through gene editing, stopping its continual deleterious effects. Second, they are trying to suppress the expression of mutated *mHTT* at the mRNA level, preventing its release at the protein level (Duan et al., 2023b; Tabrizi et al., 2022). Moreover, related research and applications, such as the investigation of the role of the DNA mismatch repair (MMR) gene *MSH3*, a genetic modifier involved in HD onset and progression, within the process of CAG expansion, have gradually unfolded. Leveraging and manipulating this gene may play a crucial role in controlling and treating HD (Carter, 2023; Driscoll et al., 2024; Flower et al., 2019; O’Reilly et al., 2023; Tomé et al., 2013) (Figs. 1 and 2).

Internationally, leading corporations such as Roche, Novartis, and PTC Therapeutics are at the forefront of pioneering gene-editing pharmaceuticals for combatting HD. These avant-garde therapeutic solutions span an array of cutting-edge modalities, from antisense oligonucleotide (ASO) and RNA interference (RNAi) therapies to small-molecule splicing regulators, zinc finger proteins (ZFPs), and the clustered regularly interspaced short palindromic repeats (CRISPR) gene-editing tools. These revolutionary treatments bring hope to those afflicted with HD, yet they are associated with their own set of intricate challenges. Currently, a series of auspicious new medications, including Tominersen, WVE-003, AMT130, Branaplam, and PTC-518, are under development across various stages of the HD therapeutic pipeline. Additionally, numerous other potential gene therapies involving animal models and *in vitro* tests are currently in the preclinical research phase, with the aim of validating their safety and efficacy before moving on to human clinical trials. As these studies progress, it is possible that the coming years will see the introduction of novel treatment methods for HD on the market (Tables 1 and 2).

**Table 1.** Pre-clinical and clinical trials for gene therapy registered at the International Clinical Trials Research Platform (ICTRP) for people with Huntington’s disease.

Drug	Registration ID	Trial name	Sponsor	Delivery	Dose	DNA or RNA targeting	Allele selectivity	Effective to exon1 protein	Status	Main outcome
ASO										
Tominersen (ISIS443139)	NCT02519036	-	Ionis Pharmaceuticals，Inc.	Intrathecal	Multiple	RNA	No	No	Phase Ⅱ	Dose-dependent reductions in concentrations of mutant huntingtin
Tominersen (ISIS443139)	NCT03342053	IONIS-HTT_RX_ OLE	Ionis Pharmaceuticals，Inc.	Intrathecal	Multiple	RNA	No	No	Phase Ⅱ	Safety and tolerability at 74 weeks
Tominersen (RG6042)	NCT04000594	GEN-PEAK	Hoffmann-La Roche	Intrathecal	Multiple	RNA	No	No	Phase Ⅰ	Pharmacodynamics and pharmacoki netics at multiple timepoints until 6 months
Tominersen (RG6042)	NCT03842969*	GEN-EXTEND	Hoffmann-La Roche	Intrathecal	Multiple	RNA	No	No	Phase Ⅲ	Safety and tolerability at up to 5 years
Tominersen (RG6042)	NCT03761849	GENERATION-HD1	Hoffmann-La Roche	Intrathecal	Multiple	RNA	No	No	Phase Ⅲ	Clinical efficacy at 101 weeks
Tominersen	NCT05686551	GENERATION-HD2	Hoffmann-La Roche	Intrathecal	Multiple	RNA	No	No	Phase Ⅱ	Safety at 24 months
WVE-120101	NCT03225833	PRECISION-HD1	Wave Life Sciences Ltd.	Intrathecal	Multiple	RNA	Yes	No	Phase Ⅰb/Ⅱa	Safety and tolerability at 1 and 120 days
WVE-120102	NCT03225846	PRECISION-HD2	Wave Life Sciences Ltd.	Intrathecal	Multiple	RNA	Yes	No	Phase Ⅰb/Ⅱa	Safety and tolerability at 1 and 120 days
WVE-003	NCT05032196	SELECT-HD	Wave Life Sciences Ltd.	Intrathecal	Multiple	RNA	Yes	TBC	Phase Ⅰb/Ⅱa	Safety at 36 weeks
VO659	NCT05822908	-	Vico Therapeutics B. V.	Intrathecal	Multiple	RNA	Possible	Yes	Phase Ⅰ/Ⅱa	Safety at 253 days
TTX-3360	-	-	Triplet Therapeutics	ICV	-	RNA	N/A	N/A	Pre-clinical	Failed
RNAi										
AMT130	NCT04120493	-	UniQure Biopharma B.V.	Intra-striatal	Single	RNA	No	Yes	Phase Ⅰ/Ⅱ	Safety at 18 months
VY-HTT01	NCT04885114	-	Voyager Therapeutics	Intraparenchymal	Single	RNA	No	No	Phase 1b	Hold placed
ALN-HTT	-	-	Alnylam Pharmaceuticals	Intra-striatal	Single	RNA	No	Yes	Pre-clinical	Alleviated the molecular and motor dysfunctions in HD mouse model
MSH3-RNAi	-	-	-	ICV	Single	RNA	N/A	N/A	Pre-clinical	Effectively blocked CAG-repeat expansion in the striatum of HD mouse models
Small molecule splicing modulator										
Branaplam	NCT05111249	VIBRANT HD	Novartis Pharmaceuticals	Oral	Multiple	RNA	No	No	Phase Ⅱ	Reduction of mHTT protein at week 17, safety at 104 weeks
PTC518	NCT05358717	PIVOT HD	PTC therapeutics	Oral	Multiple	RNA	No	No	Phase Ⅱ	Safety at 113 days
Compound 27	-	-	-	Oral	Multiple	RNA	No	No	Pre-clinical	Significantly reduced HTT levels in both HD human stem cells and HD mouse models

**Table 2.** Novel gene editing platform.

Gene editing	Delivery	DNA or RNA targeting	Allele selectivity	Effective to exon1 protein	Main outcome	Status	References
ZFP	ICV	DNA	Yes	Yes	Reduced mHTT protein aggregation, and alleviated behavioral symptoms	Pre-clinical	Garriga-Canut et al. (2012)
mZF-KRAB	ICV	DNA	Yes	Yes	Prolonged suppression of mutant HTT expression in the brain	Pre-clinical	Agustín-Pavón et al. (2016)
ZFP-TF	ICV	DNA	Yes	Yes	Reduced expression of the mHTT gene	Pre-clinical	Zeitler et al. (2019)
TALENs	ICV	DNA	Yes	Yes	Degradation of the mutant Huntington allele	Pre-clinical	Fink et al. (2016)
CRISPR/Cas9	ICV	DNA	Yes/No	Yes	Decreased or rectified the expression of the mHTT gene	Pre-clinical	Dabrowska et al. (2018), Ekman et al. (2019), Lopes et al. (2020), Monteys et al. (2017), Oikemus et al. (2022), Shin et al. (2016), Xu et al. (2017), Yan et al. (2023), Yang et al. (2017)
CRISPR/Cas13	ICV	RNA	Yes	Yes	Reduced expression of mHTT mRNA	Pre-clinical	Morelli et al. (2023)

### ASO therapy

ASO therapy has garnered considerable attention and is categorized based on its mechanisms of action into those that involve RNA degradation and those that inhibit or modulate mRNA expression through steric hindrance. In 2015, ASOs successfully entered clinical trials for the first time, with preliminary results demonstrating a significant reduction in the levels of the toxic protein mHTT in the cerebrospinal fluid (CSF) of HD patients, indicating good tolerance to ASOs and confirming their safety (Medina Escobar et al., 2019; Tabrizi et al., 2019b). However, despite the promise this therapy has shown, the reality is that multiple ASO drugs being developed for HD treatment continue to encounter research and development bottlenecks.

#### Tominersen

Tominersen, formerly known as IONIS-HTTRx and RG6042, is an antisense oligonucleotide that aims to reduce the levels of the HTT protein in the brain. It is delivered into the CSF via intrathecal injection into the spinal fluid, which surrounds the brain and spinal cord. Originally developed by Ionis Pharmaceuticals, Tominersen was licenced to Roche in 2017. Its clinical development began in 2015, and a phase II trial (NCT02519036) showed that treatment significantly reduced the levels of mHTT in the CSF of patients with early manifestations of Huntington’s chorea (Tabrizi et al., 2019b). A global phase III trial called GENERATION HD1 (NCT03761849) followed (Boak and McColgan, 2022; Tabrizi et al., 2019b, 2022), testing the safety and efficacy of Tominersen versus a placebo in 791 patients with manifest Huntington’s chorea. Participants were randomized to receive 120 mg of Tominersen every two or four months or a placebo over a period of up to two years. The trial’s primary objective was to assess changes in disease severity using the composite Unified Huntington Disease Rating Scale (cUHDRS), which assesses cognitive and motor skills and the ability to perform daily activities independently. Preliminary results, however, indicated that the treatment was less effective than a placebo, particularly in the high-dose group, which displayed a faster progression of neurologic decline, prompting Roche to cease the trial’s progression in March 2021.

There are numerous reasons for this failure, including the widespread early distribution of the drug to the leptomeninges and cortex after intrathecal injection, with subsequent gradual spread throughout the brain potentially leading to localized high concentrations that induce neuronal toxicity, with early elevated neurofilament light (NfL; a biomarker of neuronal cell damage that can reflect the impact of focal inflammatory disease) levels in the CSF as a marker for neuronal death in the high-dose group; the simultaneous reduction of both w*HTT* and m*HTT* by Tominersen is also a potential problem, although most current therapies nonspecifically degrade both; insufficient penetration of ASO drugs into the basal ganglia, a key neuroanatomical structure in HD, could also be a contributing factor (Tabrizi et al., 2019b). *Post hoc* exploratory analyses suggested that Tominersen might benefit younger patients with a lower disease burden (Boak and McColgan, 2022; Schobel, 2021).

Roche began a new phase II clinical trial, GENERATION HD2 (NCT05686551), in early 2023, aiming to explore the efficacy of Tominersen at doses of 60 and 100 mg in a population of young-adult patients with a relatively low disease burden. This study is a multicentre, double-blind, placebo-controlled trial planned to enroll 360 patients. The regimen consists of dosing every 16 weeks over a 16-month period. The primary endpoints of the research are the changes in the UHDRS and total functional capacity (TFC) score compared to baseline at the 16th month (Estevez-Fraga et al., 2023).

The primary objectives of GENERATION HD2 are to evaluate the safety and CSF levels of mHTT therapy and changes in the cUHDRS and TFC. Secondary outcomes include a variety of measures of cognitive and motor function, as well as changes in NfL levels in CSF. The generation of antibodies against the therapy will also be monitored. Like the earlier GENERATION HD1 study and other trials, a Data Monitoring Committee will review safety, clinical, and biomarker data every four to six months. GENERATION HD2 is expected to be completed in January 2027.

#### WVE-120101 and WVE-120102

Wave Life Sciences, a biotechnology company, has made progress in developing allele-selective treatments for HD via the ASOs WVE-120101 (NCT03225833) and WVE-120102 (NCT03225846) (Hersch et al., 2017). These ASOs are tailor-made to target two specific single-nucleotide polymorphisms (SNPs) related to HD gene mutations. While approximately 40% of individuals with HD of European heritage have either of these SNPs, some may carry both (Kay et al., 2014, 2015), allowing ASOs to potentially benefit a broad segment of the HD population, ranging from 36%–70% (Kay et al., 2014).

During the phase I/Ia clinical trials PRECISION-HD1 and PRECISION-HD2, doses up to 16 mg did not result in serious adverse events, and an early outcome from PRECISION-HD2 indicated a statistically significant reduction in mHTT protein in the CSF of 12.4% (Rook and Southwell, 2022). However, this reduction was modest compared to those of broader-range HD ASOs, and a significant reduction was not consistently observed in PRECISION-HD1 test subjects. Trials for higher doses (32 mg) were later initiated but ultimately halted in 2021 due to safety concerns and a lack of consistent mtHTT reduction. This higher dose resulted in serious adverse events in more than half of the participants, leading to termination of the treatment (Viglietta, 2021).

#### WVE-003

In response, Wave refocused their efforts on a new ASO, WVE-003, which targets a different SNP that could benefit nearly 40% of the HD population. Benefiting from a novel chemical structure featuring phosphoramidate (PN) modifications, WVE-003 is expected to have greater potency, greater CNS distribution, and a longer half-life than its predecessors. Prevailing preclinical studies have shown encouraging decreases in mHTT in motor neurons and rodent models, indicating a strong starting point for the therapeutic (Rook and Southwell, 2022) potential of this drug. For ongoing WVE-003 (NCT05032196) development in clinical settings, the phase 1b/2a trial SELECT-HD has provided useful data. The dose was 30 mg every eight weeks, the single dose of WVE-003 showed good safety and tolerability, and the CSF mHTT protein concentration decreased; after a single administration for 85 days, the average mHTT protein concentration in the two cohorts decreased by 22%. WVE-003 has acted as expected, selectively reducing the toxic mHTT protein while avoiding its impact on healthy wild-type Huntington’s protein, thus maintaining its beneficial role in the CNS. Additionally, in a non-human primate study, WVE-003 achieved significant tissue exposure in deep brain areas (including the striatum). These data support Wave’s existing dataset, which validates the ability of drugs to be distributed in CNS areas related to HD. Wave expects to report 30 mg multi-dose cohort data and long-term follow-up data in the second quarter of 2024, including all single-dose data.

#### VO659

Vico therapeutics is developing a treatment called VO659 (NCT05822908) for polyQ diseases like HD and spinocerebellar ataxias. This treatment selectively targets harmful CAG repeats in the genes responsible for these diseases. VO659 works by blocking harmful proteins and has shown promising results in both cell studies and mouse models, particularly in improving symptoms in juvenile HD patients with longer CAG repeats (Datson et al., 2017). VO659 has a favorable lasting effect on the brain and is more effective than a nonselective competitor in early tests. Based on strong preclinical data, VO659 has entered a phase 1/2a clinical trial, marking the first potential broad treatment for all nine polyQ diseases in clinical evaluations. Its unique targeting approach may allow for a safer and more effective treatment window (Estevez-Fraga et al., 2024).

#### TTX-3360

TTX-3360 is an ASO drug developed by triplet therapeutics for repeat expansion diseases such as HD, myotonic dystrophy, and spinocerebellar ataxias. The therapy ataxias. Therapy is administered via intracerebroventricular (ICV) injection, specifically targeting the inhibition of the key gene *MSH3* in the DNA damage response (DDR) pathway that drives repeat sequence expansion in these diseases. The strategy of triple therapy is to intervene in the DDR pathway to inhibit the onset and progression of various repeat expansion disorders (Ferguson et al., 2022).

### RNAi therapy

RNAi achieves gene silencing by introducing RNA of specific sequences into the cell, including short interfering RNA (siRNA), short hairpin RNA (shRNA), and microRNA (miRNA), to form ribonuclease silencer complexes that bind complementarily to mRNAs with specific sequences, inducing their degradation and preventing their translation into proteins (Wild and Tabrizi, 2017). Because these double-stranded RNA molecules have a relatively limited diffusion range in brain tissue, they rely on vectors to enter the brain, which is typically achieved by the intracerebral targeted injection of viral vectors. Through viral transfection, exogenous sequences can be stably expressed in target cells over the long term, allowing the cells themselves to produce precursors to siRNAs such as shRNAs/miRNAs, thus achieving gene silencing.

Research on the treatment of HD has explored two main siRNA silencing techniques. These strategies specifically target the HTT and *MSH3* genes. The *HTT* gene, when overexpressed due to the expansion of the CAG repeat sequence, leads to the production of an abnormal HTT protein that induces HD. By suppressing *HTT* gene expression using siRNA, the levels of this abnormal protein can be reduced (Caron et al., 2020; Keskin et al., 2019; Miniarikova et al., 2016, 2017; Spronck et al., 2021). Moreover, interventions are focused on the *MSH3* gene, which plays a significant role in CAG repeat sequence expansion (Tomé et al., 2013).

#### AMT-130

uniQure developed a gene therapy drug named AMT-130 (NCT04120493), which utilizes adeno-associated virus 5 as a vector for allele-specific miRNA therapy and is designed to provide a permanent effect with one-time brain surgery for the HD gene. AMT-130 stimulates the RNA-induced silencing complex, effectively halting the synthesis of both mutant (mHTT) and normal (wtHTT) HTT proteins. AMT-130 is administered through an intrastriatal injection (Evers and Konstantinova, 2020).

In 2022, the company announced phase I/II study results, which indicated that a low dose of AMT-130 appeared to be safe with limited side effects, and early signs of HTT protein reduction were observed in a few participants with available data. However, in the high-dose injection group, three individuals experienced serious neurological issues after surgery. Encouragingly, on December 2023, uniQure announced updated interim data from the treatment of HD with AMT-130, showing some positive outcomes. The exploratory efficacy data for AMT-130 revealed potential clinical benefits compared to those in a natural history cohort, with improvements or preservation in neurological function as measured by cUHDRS, TFC, and transcranial magnetic stimulation in patients, showing favorable differences at different time points for both low- and high-dose cohorts. In terms of biomarkers, mean CSF NfL levels suggest a reduction in neurodegeneration, although temporary postsurgical increases were observed and not related to dosage. mHTT levels in the CSF continued to show variable results and are not considered wholly representative due to direct brain administration. In terms of safety and tolerability, AMT-130 demonstrated good tolerability (Saade and Mestre, 2024).

#### VY-HTT01

VY-HTT01 (NCT04885114) was developed by Voyager Therapeutics and utilizes an AAV1 vector to deliver a genetic sequence into brain cells that produces a miRNA targeting *HTT* mRNA, which can decrease harmful HTT protein levels and potentially slow the progression of HD by preventing nerve cell death. Preclinical studies in animal models have yielded encouraging results, demonstrating efficient delivery to brain cells, effective reduction of HTT levels, and no significant toxic side effects (Ghilan et al., 2014; Stanek et al., 2014). This is the first clinical trial of VY-HTT01 for early-stage HD. This phase 1b study, which was conducted across multiple centers, aimed at evaluating the safety and tolerability of this therapy. The therapy is administered at four different dose levels via an infusion into two areas of the brain, the putamen and thalamus.

Currently, Voyager Therapeutics have discontinued their 1st generation HD program with the VYTAL Study and have initiated a 2nd generation program. They advanced research in gene therapy for HD using tropism redirection of AAV by cell-type-specific expression of RNA (TRACER) capsid technology to specifically target cells with AAV vectors. This approach may allow for intravenous administration and could provide widespread distribution to affected brain tissue. Their approach employs vectorized siRNAs to knock down m*HTT*, the protein causing HD, and *MSH3*, a gene implicated in disease progression. The company’s presentation at the CHDI’s 18th Annual HD Therapeutics Conference suggests progress in developing more precise treatments that may eventually provide hope for HD patients (Carter, 2023).

#### ALN-HTT

Alnylam Pharmaceuticals published promising preclinical results for the RNAi therapeutic ALN-HTT, targeting HD. The study demonstrated that direct infusion of a siRNA targeting the *HTT* gene into the striatum using convection-enhanced delivery resulted in a broad distribution and significant silencing of *HTT* mRNA, with an approximately 45% reduction in the putamen and a favorable safety profile (Stiles et al., 2012).

Currently, Alnylam has harnessed its advanced CNS delivery platform to innovate and scrutinize a 2ʹ-O-hexadecyl (C16) group linked siRNA (C16-siRNA) conjugate (Brown et al., 2022; Anonymous, 2022). This pioneering conjugate, which was tested in nonhuman primates (NHPs), delivered promising results, showing that a single intrathecal injection can produce strong and tolerable reductions in HTT at both 3 and 6 months, as evidenced by assessments of terminal tissue protein levels, CSF NfL concentrations, and histopathology. These findings underscore that the C16-siRNA platform can offer new possibilities for the treatment of HD by providing a potential new path for reducing HTT (Cantley, 2023).

#### siRNA-mediated silencing of *MSH3* (*MSH3-*RNAi)

Inheritance of expanded CAG repeats is necessary for HD manifestation, but further somatic expansion of the repeat tract in non-dividing cells, especially striatal neurons, accelerates disease onset. This process, known as somatic repeat expansion, is mediated by the MMR pathway. Among the MMR components identified as modifiers of HD onset, *MSH3* has emerged as a potentially safe and effective target for therapeutic intervention.

In recent research, a fully chemically modified siRNA was identified that robustly silenced *Msh3* both *in vitro* and *in vivo*. When synthesized in a divalent scaffold, siRNA-mediated *Msh3* silencing effectively blocked CAG repeat expansion in the striatum of two HD mouse models without affecting tumor-associated microsatellite instability or the mRNA expression of other MMR genes (O’Reilly et al., 2023). However, the extent to which MSH3 protein reduction is needed to alleviate somatic CAG repeats and elicit therapeutic effects in HD disease models remains unclear. In another recent study, researchers used potent bia-siRNAs to silence mouse *Msh3* mRNA expression in a dose-dependent manner in HdhQ111/+ mice. They then correlated somatic *Htt* CAG instability with MSH3 protein levels from simultaneously isolated DNA and protein after siRNA treatment. The results revealed a linear relationship, with a proportionality constant of approximately 1, between the prevention of somatic *HTT* CAG repeats and MSH3 protein expression *in vivo*. These findings support the role of MSH3 as a rate-limiting step in somatic CAG repeats (Driscoll et al., 2024). The results also provide important insights for developing therapeutic molecules targeting *MSH3* as potential therapeutic targets for HD.

### Small-molecule splice modulators

Small-molecule splice modulators are special sequences present in the introns of *HTT* mRNA that introduce a “pseudoexon” and generate a premature termination codon into the mature *HTT* mRNA, leading to the degradation of the mRNA and thereby reducing the expression of the pathogenic HTT protein. These orally administered small-molecule drugs can cross the blood-brain barrier (BBB), lowering *HTT* mRNA levels in the CNS as well as peripherally.

#### Branaplam (LMI070)

In the field of HD, the development of small-molecule splice modulators has been fraught with challenges. Branaplam (LMI070), a therapeutic candidate manufactured by Novartis Pharmaceuticals, is an orally administered molecule that has shown potential in early clinical trials (Palacino et al., 2015). It modifies the mRNA splicing of the *HTT* gene, introducing a pseudoexon that triggers mRNA degradation and consequently reducing the expression of the pathogenic HTT protein (Keller et al., 2022).

However, during the progression of the VIBRANT-HD clinical trial (NCT05111249), which was designed to evaluate the safety, tolerance, pharmacodynamics, and pharmacokinetics of weekly oral administration of Branaplam in patients diagnosed with early-stage HD, a pause in dosing was announced in August 2022 due to peripheral neuropathy symptoms emerging in several study participants. Subsequently, in December of the same year, Novartis decided to halt the development of Branaplam as a potential treatment for HD. Insights were obtained from nonblinded follow-up data from the VIBRANT-HD study, which revealed a negative benefit–risk ratio for Branaplam in HD treatment (Estevez-Fraga et al., 2024) .

#### PTC518

The development of PTC therapeutics is progressing proactively through the development of an oral small-molecule splice modulator, PTC518, which has the potential to treat HD. Recruitment is currently underway for the phase II PIVOT-HD trial (NCT05358717), which is studying this promising therapeutic candidate.

Preliminary data from the PIVOT-HD trial have generated optimism, indicating both a good tolerability profile for PTC518 and its efficacy in reducing levels of problematic HTT in afflicted patients. Critically, no sudden surges in NfL levels have been observed, suggesting minimal nerve cell injury. This encouraging step in the study represents a significant positive development for patients combating HD and provides a beacon of hope for the broader medical community. As we move forward, the anticipation surrounding additional data and prospective findings from the PIVOT-HD study continues to build (Beers et al., 2022)

#### Compound 27

In addition to the above-mentioned clinical drugs composed of small-molecule splice modulators, recent research has designed a series of *HTT* pre-mRNA splicing modulators that decrease the harmful levels of HTT by inserting a pseudoexon at the junction of exons 49–50 of the *HTT* gene. This pseudoexon causes the transcripts to degrade, significantly reducing the quantities of *HTT* mRNA and protein produced. Among them, Compound 27 is particularly effective, significantly lowering HTT levels in both human HD stem cells and HD mouse models. Similar to the aforementioned small-molecule splicing modulators, Compound 27, which has been optimized, shows good CNS penetrability and oral therapeutic properties. Furthermore, this study may provide insights into some side effects associated with Branaplam treatment, such as peripheral neuropathy. By studying the mechanisms of these modulators, it may be possible to find ways to mitigate these side effects (Liu et al., 2023).

The application of small molecule splicing modulators in the treatment of HD has brought new hope. These drugs can reversibly modulate disease-associated genes without interfering with the genome. Furthermore, compared to RNAi delivered via viral vectors or ASOs requiring repeated intrathecal injections, orally administered small molecule splicing modulators offer clear advantages in terms of administration (Keller et al., 2022). However, hope comes with its own set of challenges. Given that the HTT is widely expressed in both the brain and peripheral tissues and is involved in various cellular processes, the long-term safety of non-allele-selective HTT reduction remains a concern (Kaemmerer and Grondin, 2019). A recent study reported four compounds that target mHTT protein for autophagic degradation, thereby reducing its levels in an allele-selective manner and rescuing disease-relevant phenotypes in cell, Drosophila and mouse models of HD (Li et al., 2019). Consequently, for small molecule splicing modulators, more preclinical research is needed to optimize their tissue-targeting capabilities, ensuring that these drugs can effectively reach their targets in the CNS while minimizing exposure to peripheral tissues. We eagerly anticipate future research overcoming these challenges, paving the way for new directions in the treatment of HD and related neurodegenerative disorders.

### Novel gene editing platforms

Novel gene-editing strategies, including the use of Zinc finger proteins (ZFPs) transcription activator-like effector nucleases (TALENs) and CRISPR-associated protein systems (Cas), aim to reduce mHTT expression. Such innovative approaches are under investigation for their potential to modify the disease course at the molecular level.

#### ZFPs

ZFPs can recognize and bind to specific DNA sequences, and this capability is not static. These DNA recognition properties can be experimentally adjusted, making ZFPs exceptionally versatile and suitable for generating precise genetic modifications. Moreover, the combination of zinc finger nucleases (ZFNs) and the cleavage domain of Fok I restriction endonuclease forms an impressive tool for genome editing. ZFNs benefit from the Fok I endonuclease by gaining the capacity to induce site-specific double-strand breaks (DSBs) in DNA (Kim et al., 1996).

Using a “molecular tape measure”, Garriga-Canut et al. developed synthetic ZFPs that bind strongly to long sequences of CAG repeats. This optimized ZFP significantly reduced the expression of mutated genes in HD model cells without affecting the expression of shorter wild-type alleles, other genomic CAG-repeat genes, or adjacent genes. Experiments were also conducted on animals in which synthetic ZFP, delivered via AAV to the brains of R6/2 mice, decreased the expression of the m*Htt* gene by 60%. ZFP also displayed dose-dependent effectiveness, reduced protein aggregation, and alleviated behavioral symptoms in R6/2 mice, demonstrating the potential of using synthetic transcription factor inhibitors to decrease HTT expression in the brain in the context of HD (Garriga-Canut et al., 2012).

Agustín-Pavón et al. subsequently designed a host-matched version of a synthetic ZFP to target expanded DNA CAG repeats. This version, called mZF-KRAB, did not trigger significant inflammatory responses or neuronal loss when delivered using an rAAV vector, unlike the previous non-host-matched version, ZF-KOX1. Through the use of a nonviral neuron-specific enolase promoter, mZF-KRAB resulted in significantly longer-lasting repression mHTT expression in the brain (Agustín-Pavón et al., 2016). These findings hinted at the potential of mZF-KRAB and its delivery method for HD treatment.

Additionally, a team from Sangamo Therapeutics developed a strategy using their ZF Genome Engineering gene regulation technology platform, which includes a zinc finger protein transcription factor (ZFP-TF) configured to selectively target and reduce pathological CAG repeat mHTT protein levels. Using neurons and fibroblasts sourced from patients, the team demonstrated that ZFP-TFs could selectively suppress more than 99% of HD-causing mutant alleles across a broad dose range while preserving the expression of more than 86% of normal alleles. By administering ZFP-TFs to HD neurons via AAV, they demonstrated sustained activity and high tolerance, with the ZFP-TFs remaining effective for over 100 days in culture and persisting for a minimum of nine months in mouse brains. Using three different HD mouse models, this study revealed improvements across various molecular, histopathological, electrophysiological, and functional areas (Zeitler et al., 2019). These groundbreaking discoveries provide support for the continuous research and development of allele-selective ZFP-TFs as potential treatments for HD. These impressive results provide strong preclinical support for the use of the Sangamo Therapeutics ZFP-TF gene regulation technology platform as a promising new therapeutic avenue for HD.

#### TALENs

Transcription activator-like effectors (TALEs) are proteins that were initially discovered in a bacterium called *Xanthomonas*. They can recognize and bind to specific DNA sequences, thereby modulating gene expression. In the context of gene editing, TALEs exhibit great advantages in terms of specificity. Pairing TALEs with a nuclease domain capable of inducing DSBs in DNA, such as the FokI nuclease, results in the creation of TALENs. TALENs can precisely bind and cleave specific DNA sequences, making them highly effective tools for targeted gene editing (Zhang et al., 2014).

A pioneering preclinical study probed the use of TALEs for allele-specific degradation of the mutant Huntington allele in human HD fibroblasts. Researchers engineered TALEs to target SNPs in the mutant allele and utilized a vector with a krüppel-associated box (KRAB) effector to promote transcriptional repression of this disease-related allele. Additional TALEs were engineered as nuclease TALENs to induce collapse of the CAG repeats of the mutant allele. This study demonstrated that the application of TALEs might substantially reduce the expression of the mutant allele without significantly impacting the nonmutant expression. This finding proves the viability of using TALE proteins for allele-specific gene modifications, paving the way for targeted therapies for HD and other genetic disorders (Fink et al., 2016).

This exciting study shows that, if successful in clinical trials, it could offer new possibilities for treating genetic diseases such as HD. However, more research is required to ensure safety and effectiveness before the clinical stage can be reached, and technical challenges must be addressed, including enhancing TALEN specificity, managing the immune response, and achieving precise CNS delivery.

#### CRISPR-Cas systems

The simplicity and efficiency of the CRISPR system make it an attractive option for biomedical research and disease treatment. In therapeutic strategies for HD, the application of CRISPR-Cas9 technology may aim to eliminate expanded CAG repeats to rectify the HD-associated allele, deactivate the allele linked to HD, or even directly target the *HTT* gene, with the effect of reducing HTT levels indiscriminately.

**Figure 3.**
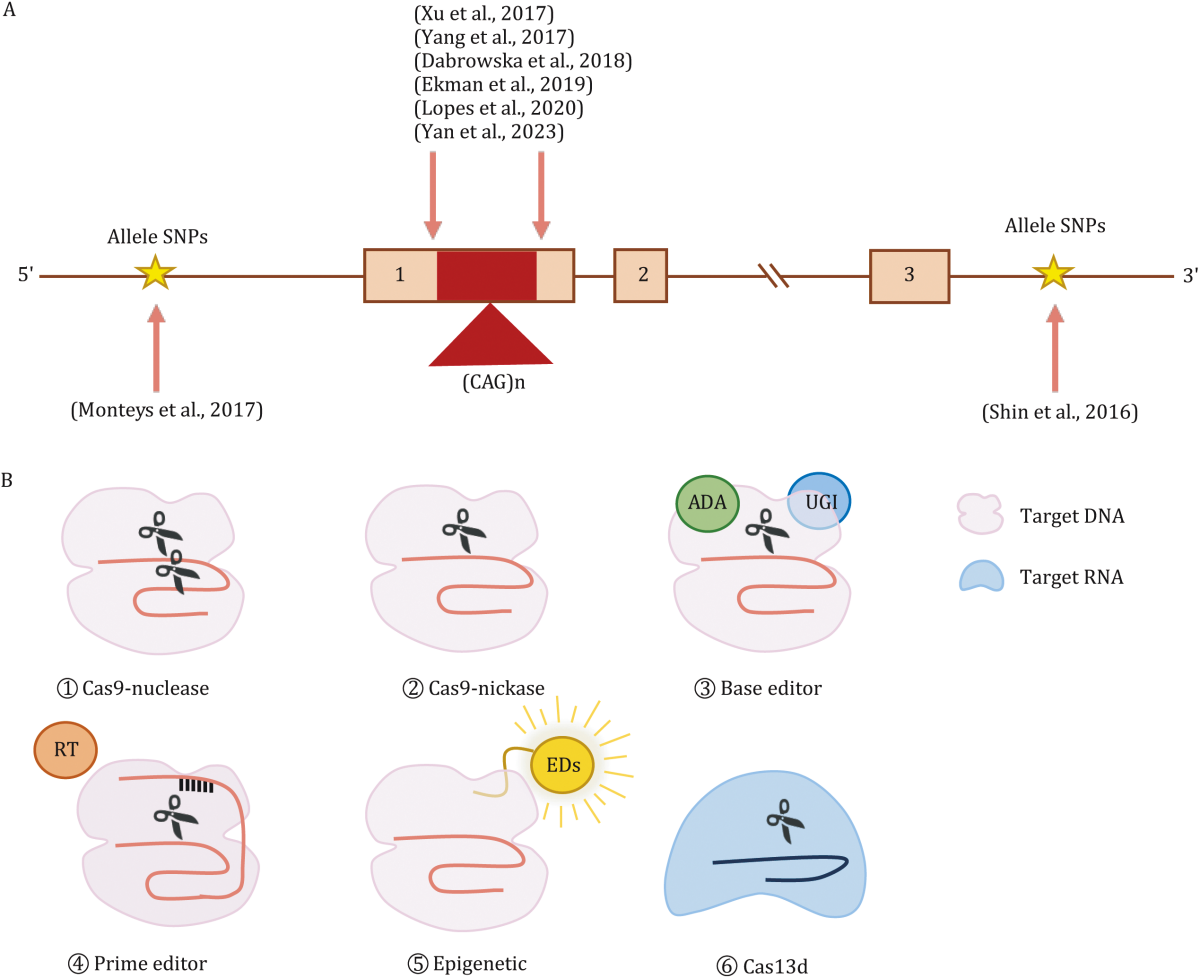
Editing approaches for treating Huntington’s disease. (A) Illustrates that different HD patients carry varying lengths of CAG repeats within the *HTT* gene. The red boxes represent the different lengths of CAG expansion mutations, and the yellow stars indicate SNPs. (B) Depicts various gene editing tools, including ① Cas9-based nuclease, ② Cas9-nickase, ③ base editor, ④ prime editor, ⑤ epigenetic modifications and ⑥ Cas13d.

To date, in addition to nonallele *mHTT* targeting (Ekman et al., 2019), the CRISPR/Cas9 system has been utilized for selectively inactivating *mHTT* genes by targeting protospacer adjacent motif sites generated by SNP alleles (Monteys et al., 2017; Oikemus et al., 2022; Shin et al., 2016). Furthermore, a nonallele-selective method has been employed to silence the *HTT* gene using a pair of single guide RNA (sgRNAs) flanking CAG repeats and Cas9 in an HD transgenic mouse model (Yang et al., 2017) or in cells derived from HD patients (Lopes et al., 2020). Nonalleleselective suppression of *HTT* gene expression has also been achieved through the CRISPR interference strategy (CRISPRi) in HEK293T cells. Additionally, the paired Cas9 nickase strategy has been demonstrated to inactivate the *HTT* gene by targeting sequences directly flanking the CAG repeat tract, resulting in the abrogation of protein synthesis in fibroblast cell lines derived from HD patients (Dabrowska et al., 2018). Moreover, methods such as paired nCas9 and piggyBac transposase strategies have been investigated for correcting mutations in induced pluripotent stem cells (iPSCs) and fibroblasts obtained from patients with HD (Xu et al., 2017) (Fig. 3A).

Given the closer resemblance of large animal models to human brain structure and function, Yan et al. established an HD knock-in (KI) pig model exhibiting the characteristic neurodegeneration and motor dysfunction typical of HD (Yan et al., 2018). Utilizing this HD-KI pig model, researchers applied both brain and intravenous injections of AAV9*-HTT*-gRNA-20Q/Cas9. They demonstrated that a single dose administered via these two delivery routes could effectively decrease the expression of m*HTT* with a degree of replacement by wt*HTT*, subsequently decreasing neuropathological changes and alleviating neurological symptoms. Furthermore, this study comprehensively assessed the safety profile of this therapeutic approach (Yan et al., 2023).

Employing CRISPR-Cas9 gene-editing technology comes with the inherent risk of off-target effects that could lead to permanent or hereditary alterations (Guo et al., 2023). Consequently, numerous studies have shifted their focus towards CRISPR systems that target RNA as an alternative that does not permanently modify the genome (O’Connell, 2019; Zhang et al., 2018). Using CRISPR-Cas13d, Morelli et al. elegantly developed a new therapeutic strategy. Their designed CAG^EX^-targeted Cas13d system (Cas13d–CAG^EX^) efficiently targets and reduces the levels of aberrant CAG-expanded *HTT* RNA in neurons derived from individuals with HD, adeptly addressing CAG repeat expansions within a range of 66 to 109 short tandem repeats. AAV-mediated delivery of Cas13d–CAG^EX^ to the striatum of premanifest zQ175/+ HD mice resulted in allele-selective suppression of m*HTT* mRNA and protein aggregates while maintaining normal *HTT* mRNA and protein levels, significantly improved motor function and attenuated striatal atrophy (Morelli et al., 2023). This discovery offers new hope for the treatment of HD. However, this research is still at the stage of laboratory, and further research and clinical trials are needed to validate its safety and efficacy.

Some studies have demonstrated that the age of onset of HD is primarily determined by the length of uninterrupted CAG repeats (Ciosi et al., 2019; Wright et al., 2019). Choi et al. utilized a combination of cytosine base editors and guide RNAs (gRNAs) to introduce CAA interruptions within the CAG repeat sequence of the *HTT* gene to reduce the length of uninterrupted CAG repeats. This base editing strategy efficiently converts CAG to CAA at various positions within the repeat sequence without causing significant insertions, deletions, off-target edits, or transcriptomic changes, demonstrating the feasibility and specificity of the strategy. In applications with the HD-KI mouse model, this strategy not only preserved the *HTT* mRNA and protein levels but also significantly reduced somatic CAG repeat expansion, which is a major driver of HD progression. Notably, in HD-KI mice with CAA interruption, the expansion of CAG repeats was completely abolished (Choi et al., 2023).

This study conducted a proof-of-concept exploration of cytosine base editing technology for the first time, demonstrating its potential as a treatment method for HD and other repeat expansion diseases with similar mechanisms. Compared to ASO or siRNA treatment approaches, base editing strategy could correct the mutation at DNA level, and avoids double-stranded breaks. However, assessing the off-target effects of base editing by verifying whether predicted off-target sites undergo gene editing, as described in the article, has limited persuasive power for clinical applications. Additionally, low *in vivo* target site editing efficiency restricts the clinical translation of base editing for HD. More preclinical research is needed to optimize base editing strategies and develop effective brain delivery methods. Most importantly, this study provides limited critical data and fails to sufficiently demonstrate that this technology can effectively alter disease progression. This may be due to the lack of relevant HD animal models carrying uninterrupted adult-onset CAG repeats, which are crucial for measuring the level of disease modification.

### The challenges of gene therapy

For HD, the primary goal of gene therapy strategies is to reduce the expression levels of the expanded mutation of the *HTT* gene, which typically involves early intervention at the DNA or RNA level. Various therapeutic mechanisms are under exploration, all aimed at mitigating mHTT toxicity. Promising advancements in drug development, such as the use of orally administered small-molecule splice modulators, have successfully addressed the challenges related to drug delivery and crossing the BBB. However, persistent issues remain, such as insufficient specificity for m*HTT* and safety concerns requiring further investigation (Tabrizi et al., 2020). Moreover, the necessity for routine intrathecal or lateral ventricle injections of ASO and siRNA drugs leads to disproportionately high local drug concentrations and insufficient drug levels at the target site, posing a significant obstacle to clinical application (Boak and McColgan, 2022; Schobel, 2021). Furthermore, double-strand breaks of DNA induced by CRISPR-Cas9 editing can result in irreversible off-target events, presenting a barrier to the clinical implementation of gene therapy (Tabrizi et al., 2019a). Commonly used viral vectors for gene therapy, such as AAV, can trigger immune responses, often hindering repeated dosing (Tabrizi et al., 2022). Additionally, the above gene-editing drugs targeting the region downstream of *HTT* mRNA exon 1 are unable to effectively reduce the amount of toxic N-terminal fragments (Fig. 2). Finally, for late-stage patients with severely damaged or deceased neurons, the benefits of these therapeutic approaches may be severely limited (Table 3). Therefore, in addressing these challenges of gene therapy, the following issues warrant our attention:

**Table 3.** Advantage and disadvantage of therapeutic strategies for Huntington’s disease.

Therapeutic strategies for HD	Gene therapy		Cell therapy	
	Advantage	Disadvantage	Advantage	Disadvantage
Therapeutic target	Precision interventions target the root cause	Irreversible off-target events	Cell replacement, growth factor release and immune modulation	Challenging to seamlessly integrate with the neural network within the host brain
Therapeutic specificity	Convenient target design, predictable off-target sites, and SNPs enable allele selection	SNPs cannot address all patients	Stem cells and precursor cells have the capability to differentiate into MSNs.	The limited homing-directed differentiation ability, tumorigenesis
Delivery strategy	Intrathecal injection and lateral ventricle administration: crossing the BBB; brain parenchymal delivery: enhances drug concentration at striatum	Limited cerebrospinal fluid penetration ability, localized high concentrations but not in disease affected area; surgical risks	Cells delivery within brain parenchyma via neurosurgery stereotactic	Surgical risks, low cell survival rate after transplantation
Delivery system	Viral vector AAV: nonpathogenic, nonintegrated; nonviral vectors: low immunogenicity	Viral vectors: cause immune response, uneven tissue distribution; nonviral vectors: low delivery efficiency	Convenient optimization design of conveying devices and schemes	Cell deposition, reflux, and apoptosis
Therapeutic efficacy	Potential long-term efficacy	The efficacy in late-stage patients with extensive neuronal loss is limited	Cell replacement in advanced patients with extensive neuronal loss	Limited therapeutic efficacy

#### Optimized delivery systems

The successful transfer of exogenous genes is crucial for the efficacy of gene therapy. Strategies for gene delivery are mainly composed of three types of vectors: viral vectors, nonviral vectors, and hybrid vectors combining features of both. To date, a variety of viral vectors have been extensively explored in clinical trials and therapeutic studies involving gene delivery. However, viral vectors still face a range of challenges, such as immunogenicity, cytotoxicity, and potential pathogenicity, which restrict their application as gene delivery tools. Currently, nonviral vectors are considered the emerging frontier in gene therapy, with the development of biomaterial-based non-viral gene delivery systems steadily progressing. Nanomaterials, a major type of non-viral vector in preclinical studies, can be further divided into various categories, including lipid nanoparticles (LNPs), polymeric nanoparticles, inorganic nanoparticles, and cell membrane-based nanoparticles (Eygeris et al., 2022). LNPs are widely used in CNS diseases. They can be designed with a single or multiple phospholipid bilayers to encapsulate hydrophilic or lipophilic drugs, thereby protecting drug contents from systemic degradation and improving the bioavailability of poorly water-soluble drugs. Small-diameter LNPs can penetrate the BBB efficiently and ensure brain cell uptake through passive diffusion and transport across mucosal epithelia, showing immense potential in delivering therapeutic agents to the CNS. For CNS diseases, nanoparticle delivery routes include conventional intravenous (blood-to-brain) and oral (gut-to-brain) administration, as well as intranasal (nose-to-brain) delivery. Intranasal administration is a noninvasive method with less trauma, reduced risk of drug leakage, and prevention of gastrointestinal reactions and first-pass effects (Cooke et al., 2016; Ferreira et al., 2021). Besides delivering DNA and RNA gene-editing drugs, LNPs can efficiently deliver biomolecules and natural products for clinical purposes (Duan et al., 2023a). Multiple studies have utilized nanoparticle-based delivery systems for the treatment of HD (Birolini et al., 2021; Cong et al., 2019; Debnath et al., 2017; Wahyuningtyas et al., 2021). Additionally, cell membrane-based nanomaterials, such as exosomes, have shown effective delivery of miRNA (Lee et al., 2017) and siRNA (Didiot et al., 2016) in HD mouse models and their primary neurons, mediating efficient silencing of HTT mRNA. Although nonviral vectors have shown potential in terms of safety, ease of production, and functional diversity, they still exhibit limitations in terms of delivery efficiency, duration of gene expression, and expression levels (Agarwal, 2023). To overcome these limitations, research into hybrid vectors that combine the attributes of viral and nonviral vectors is underway to reduce immune reactions caused by viruses and to extend the durations of gene delivery and expression (Duan et al., 2021).

Therefore, developing efficient vectors capable of gene transfection both *in vitro* and *in vivo* that target the CNS is an urgent matter (Sung and Kim, 2019). The development of nonviral vectors may focus on the refinement of gene delivery systems based on biomaterials. The goal is to design vectors with excellent pharmacokinetic properties that can evade surveillance by macrophages and the immune system, penetrate and target specific tissues effectively, and guide high-quality gene expression.

#### Improvement in the development of novel gene editing platforms

Genome editing has emerged as a transformative force in life sciences and human medicine, granting unprecedented opportunities to dissect complex biological processes and address genetic diseases at their roots. CRISPR technology, which is celebrated for its exceptional efficiency and programmability, stands at the vanguard of this revolution. The efficacy and safety of CRISPR-Cas9 are pivotal elements to weigh in its clinical implementation. The unintended off-target effects of CRISPR-Cas9 could have serious implications in clinical settings. To address this concern, researchers have focused on improving the specificity of CRISPR-Cas9 by developing various CRISPR-Cas9 variants that reduce the potential for off-target effects while maintaining high efficiency. This constant innovation aims to enhance the precision and safety of genome-editing tools for therapeutic use (Anzalone et al., 2019, 2020; Huang et al., 2021; Liu et al., 2022; Nakamura et al., 2021; Ran et al., 2013) (Fig. 3B). The scope of CRISPR-Cas9 applications has expanded beyond gene editing to encompass precise genome and epigenome modifications in human cells. Researchers are exploring the manipulation of noncoding genomes, the regulation of gene pathways, and the introduction of specific gene mutations using CRISPR-Cas technologies (Liu et al., 2022). This shift towards genome engineering underscores the versatility and potential of CRISPR-based approaches in understanding and modifying genetic information. In moving towards comprehensive clinical applications, it is essential to deepen our understanding of genetic diseases such as HD while advancing gene editing technologies to be more refined and reliable. By leveraging the latest advancements in CRISPR-Cas technologies and continuously improving its precision and safety profiles, the field is poised to realize the full potential of genome editing in clinical practice.

## Cell therapy for HD

### Treatment based on fetal cell transplantation

As HD progresses, late-stage intervention may involve the replacement of lost neurons. Cell transplantation offers a viable strategy, as it is the only method that restores atrophied tissue by implanting cells capable of taking over certain functions of degenerated cells (Tabrizi et al., 2019a). The role of cell therapy primarily lies in restoring the structure and function of the striatum by supplementing cell numbers or through the secretion of neuroprotective factors, thus achieving therapeutic purposes (Jain, 2009).

In patients with HD, the MSNs located in the striatum are the most severely impacted. Regrettably, this neural loss begins in the early stages, even before the onset of clinical symptoms, and worsens as the disease progresses, effectively contributing to the trajectory of the patient’s condition (Tabrizi et al., 2012). Similar to other neurodegenerative diseases, HD triggers persistent striatal atrophy and may additionally impact other brain regions, particularly the cerebral cortex. The therapeutic efficacy of striatal transplantation alone has sparked medical debates (Douaud et al., 2006; Precious et al., 2017). To date, no effective alternatives have been discovered to restore neuron loss due to the progression of HD. Hence, cell transplantation surgery has potential for alleviating patients’ clinical symptoms.

Over the past few decades, ~70 HD patients globally have undergone experimental fetal cell transplantation procedures. This procedure involves the transplantation of neural crest cells from human fetuses into the brains of these HD patients (Table 4). A clinical trial conducted in Creteil, France, tracked four patients who exhibited considerable enhancements in cognitive and motor functions six years after transplantation (Bachoud-Lévi et al., 2000, 2006; Gaura et al., 2004). However, two other patients continued to show declines over time. Between 2000 and 2003, the “NEST-UK” research group performed transplants of bilateral human fetal striatal tissue into five HD patients. A follow-up period of six months after surgery showed no signs of tissue overgrowth or disease exacerbation, indicating the safety of the procedure (Maxan et al., 2018; Rosser et al., 2002). Long-term follow-up of patients 3–10 years postsurgery revealed no significant differences in cognitive or motor functions between transplanted and nontransplanted patients, and positron emission tomography scans did not reveal significant differences in the size of the striatal transplant tissues, supporting the reliability of allogeneic transplantation of fetal striatal cells in terms of safety (Barker et al., 2013). MIG-HD, the largest-scale randomized multicentre phase II clinical trial, also employed human fetal neural crest cells for striatal transplantation in HD patients. Assessment of the therapeutic effects 32 months postoperation did not reveal meaningful improvements in motor skills, and severe adverse events potentially correlated with transplant rejection were noted (Bachoud-Lévi and Group, 2020).

**Table 4.** Clinical trials for stem cell therapy registered at the International Clinical Trials Research Platform (ICTRP) for people with Huntington’s disease.

Drug	Registration ID	Trail name	Sponsor	Delivery	Main outcome	Dose	Status	Location
Fetal cell therapy								
FGE	NCT03119246	CAPIT-HD2	Assistance Publique	Intravenous	No study results posted	Single	-	France
Cell suspensions of FGE	ISRCTN36485475	-	The Wellcome Trust(UK)	Intravenous	Unilateral striatal tissue transplantation in HD patients is safe, but efficacy needs extended follow-up with more patients	Single	-	UK (multicenter)
FGE	NCT00190450	MIG-HD	Assistance Publique	Intravenous	No clinical benefit was found in this trial	Single	Phase Ⅱ	France (multicenter)
FSC	ISRCTN52651778	TRIDENT	Cardiff University	Intravenous	Safety and at 4 weeks	Single	Phase I	UK
Stem cell therapy								
NestaCell® (Cellavita)	NCT02728115	SAVE-DH	Azidus Brasil	Intravenous	Safety at 5 years	Single	Phase I	Brazil
NestaCell® (Cellavita)	NCT03252535	ADORE-DH	Azidus Brasil	Intravenous	Efficacy at 120 days	Multiple	Phase Ⅱ	Brazil
NestaCell® (Cellavita)	NCT04219241	ADORE-EXT	Azidus Brasil	Intravenous	Efficacy and safety at 2 years	Single	Phase Ⅱ/Ⅲ	Brazil
NestaCell®	NCT06097780	STAR	Azidus Brasil	Intravenous	Efficacy at 1 years	Multiple	Phase Ⅲ	Brazil

Overall, allogeneic transplantation of fetal striatal cells is considered safe. However, despite its safety, the efficacy of this procedure has not been proven, and it has not fully restored striatal function. Moreover, the application of fetal-derived cells in transplantation therapy presents several challenges and limitations, including ethical concerns, non-renewability of resources, and issues pertaining to quality control.

### Therapy based on neural stem cells (NSCs) or neural progenitor cells (NPCs)

Therapy based on NSCs/NPCs, a promising and burgeoning strategy, has attracted significant attention from the scientific community. These treatments aim to replace neurons that have been damaged or lost, occasionally incorporating gene correction strategies in addition to cell therapy. Stem cell-based therapies have potential advantages over traditional fetal cell transplants, such as circumventing ethical issues, mitigating the scarcity of cell sources, and offering more precise treatment control (Bachoud-Lévi et al., 2021).

Recent studies suggest an escalating trend towards the use of NSCs in the treatment of HD (Table 5). This increase can be attributed to the encouraging potential of NSCs in disease treatment. In particular, their ability to differentiate into neurons and glial cells plays a pivotal role in their emerging prominence. NSCs can be induced by various methods, including direct sourcing from the brain or conversion from somatic cells of HD patients. Although stem cell-based therapies are mainly in the early stages of preclinical and clinical trials, existing evidence indicates the therapeutic benefits of stem cells and their derivatives in the treatment of HD animal models, showing promising results (Sivandzade and Cucullo, 2021). Early studies of stem cell therapy focused on transplanting embryonic stem cell (ESC)-derived NSCs into HD animal models, revealing successful integration of these new motor neurons and their networks within the host brain.

**Table 5.** Main pre-clinical cell therapies for Huntington’s disease.

HD/Animal model	Type of cells	Pretreatment	Therapeutic effect	References
NSC/NPCs transplant				
N171-82Q mice	NPC	KI GDNF	Reduced neuron death and improved motor function	Ebert et al. (2010)
Rat model	iPSC-NSC	-	Patient-derived cells that were transplanted exhibited signs of HD pathology at later stages	Jeon et al. (2012)
R6/2 mice	iPSC-NSC	Gene correction	Gene correction reversed the disease phenotype, and NSCs can differentiate into neuron *in vivo*	An et al. (2012)
Mouse model	iPSC-NPC	Gene correction	Gene correction reversed the disease characteristics, and the transplantation of NPC improved motor function	Cho et al. (2019), Park et al. (2022), Xu et al. (2017)
QA rat	hESC-striatal progenitors	-	Striatal progenitors can differentiate into MSN and form loop connections with surrounding tissue	Schellino et al. (2023)
Neurogliocyte transplant				
R6/2 mice	hGPC	-	Reversed the disease phenotype, slowed disease progression, and prolonged survival of diseased mice	Benraiss et al. (2016)
YAC128 mice	Astrocytes	KI NeuroD1 and Dlx2	The transformed astrocytes exhibited the electrophysiological characteristics of GABAergic neurons and improved lifespan of HD mice	Wu et al. (2020)
MSC transplant				
N171-82Q mice	MSC	-	Increased neurogenesis and decreased atrophy of the striatum	Snyder et al. (2010)
YAC128 mice	hBM-MSC	KI BDNF or NGF	Reduced neuronal loss in the striatum and alleviated behavioral deficits	Dey et al. (2010)
QA rat model	hBM-MSC	KI BDNF	Improved motor function	Sadan et al. (2012b)
N171-82Q mice	hBM-MSC	Lithium and valproic acid	Enhanced the motor ability, reduced the loss of striatal neurons and HTT protein aggregates	Linares et al. (2016)
R6/2-J2 mice	hBM-MSC	-	Prolonged survival and ameliorated motor deficit of HD mice	Lin et al. (2011), Sadan et al. (2012a)
Rat model	hIDPSC	-	Restored the expression of BDNF, DARPP32, and D2R, thereby promoting neuroprotection and neurogenesis	Wenceslau et al. (2022)

Transplants of NSCs/NPCs mainly originate from two sources: direct isolation from within the animal body and derivation from *in vitro* cultured stem cells. Ebert et al. reported that unmodified NPCs transplanted directly did not exert substantial neuroprotective effects on the rodent brain. However, genetically modified NPCs expressing the neurotrophic factor glial cell line-derived neurotrophic factor (GDNF) demonstrated protective effects on neurons and facilitated functional rehabilitation (Ebert et al., 2010). These findings highlight the potential benefits and efficacy of genetically modified NPCs in therapeutic strategies.

Numerous investigations have documented the use of NPCs derived from iPSCs for HD cell replacement therapy. Jeon et al. transplanted NPCs carrying 72 CAG repeats derived from human iPSCs (hiPSC-NPCs), which were created from HD patients, into HD rat models for disease examination. Even with the reduced neural induction efficiency of HD-iPSCs carrying 72 CAG repeats, these cells were capable of differentiating into GABAergic striatal neurons—the type of neurons most prone to degeneration in HD. Researchers transplanted neural precursors derived from HD-iPSCs into a rat model of replicating HD symptoms and observed significant behavioral recovery in the grafted rats. Although no aggregate formation was detected either *in vitro* or at 12 weeks post-grafting, signs of HD pathology appeared after treatment with a proteasome inhibitor (MG132) or upon examination of cells engrafted into neonatal brains at 33 weeks post-implementation (Jeon et al., 2012). This study revealed that HD-iPSCs, with their unique characteristics, have potential for use in studying HD pathology and developing new treatments.

In another study, An et al. conducted genetic correction on iPSCs derived from HD patients (HD-iPSCs), replacing expanded CAG repeats with normal sequences via homologous recombination. This correction persisted in differentiated DARPP-32-positive neurons both *in vitro* and *in vivo* (An et al., 2012). Furthermore, the correction normalized pathological HD signalling pathways and reversed disease characteristics, such as susceptibility to cell death and alterations in mitochondrial bioenergetics (Xu et al., 2017). The generation of patient-specific iPSCs with genetic correction from HD patients will provide disease models with identical genetic backgrounds, which is crucial for ultimately using these cells in cell replacement therapy. Similarly, other studies employing a combination of stem cell technology and gene editing have shown that transplanting NPCs derived from corrected iPSCs can significantly improve the motor function of HD mice (Cho et al., 2019; Park et al., 2022). Hence, NSCs and NPCs are considered ideal cell types for treating HD due to their potential to replace damaged neural cells and correct genetic errors.

ESCs and iPSCs are produced approximately one week after fertilization and can develop into various tissues and organs. Under *in vitro* conditions, these stem cells can be directed to differentiate into neural cells such as neurons and astrocytes. Schellino et al. explored the use of hESC-derived striatal progenitors as potential treatments for HD. Striatal progenitor transplants within the rat brain survived for up to 6 months and exhibited morphological and neurochemical characteristics similar to those of MSNs. The grafts made connections with the host and improved the motor deficits caused by HD. Furthermore, enrichment conditions (EEs) can promote differentiation into MSNs and facilitate their integration (Schellino et al., 2023). These results support the potential of transplanting ESC-derived striatal progenitors for the long-term treatment of HD.

### Neuroglial cell transplantation-based therapy

In HD, degenerative changes impact a wide variety of brain cells. Not only are neurons damaged but neuroglial cells (i.e., astrocytes and oligodendrocytes) also undergo alterations in the early stages of the disease. To fully address these cellular damages, researchers have focused on the replacement therapy of neuroglial cells as a potential route for cell therapy (Benraiss et al., 2016; Rosser et al., 2022). Human glial progenitor cells (GPCs), which can differentiate into astrocytes and oligodendrocytes and exhibit strong migratory potential, are at the center of this research. In the case of HD patients, astrocytes are implicated in synaptic pathological changes and contribute to inflammatory responses (Table 5). In the research by Benraiss et al., the transplantation of healthy neural GPCs into the brains of transgenic mice carrying the HD gene demonstrated that this treatment effectively restored the physiological functions of MSNs, further leading to significant improvement in the disease phenotype of HD mouse models. These improvements included electrophysiological and behavioral phenotype mitigation, potassium ion balance restoration, disease progression deceleration, and lifespan extension in these mice (Benraiss et al., 2016).

Despite the beneficial impact of GPCs on the glial component of HD, they cannot replace lost MSNs. Therefore, researchers are exploring alternative avenues with the hope of replenishing damaged neurons by directly converting astrocytes into neurons *in vivo* or *in vitro* by activating certain specific pathways. This innovative method for neural regeneration as a treatment for HD sidesteps issues such as immune rejection and the necessity for reprogramming steps to achieve pluripotency (Chen et al., 2020; Tai et al., 2020). Several transcription factors associated with direct astrocyte reprogramming, such as NEUROD1, SOX10, NEUROG2, ASCL1, NR4A2, have been identified (Talifu et al., 2023). In studies by Wu et al., ectopic expression of the transcription factors NEUROD1 and DLX2 mediated by AAV successfully reprogrammed striatal astrocytes into GABAergic MSNs in R6/2 and YAC 128 HD mouse models, with conversion rates of up to 50%. These reprogrammed astrocytes exhibited electrophysiological properties of GABAergic neurons, and the treated mice displayed an extension of lifespan and improvements in motor functions (Wu et al., 2020).

However, unresolved controversies remain in the field of astrocyte-to-neuron conversion. In addition to findings from HD mice model, other studies have confirmed that overexpression of the *NeuroD1* gene can induce astrocytes in the brain to transdifferentiate into neurons, thereby improving phenotypes in various models of brain injury and neurodegenerative diseases (Chen et al., 2020; Guo et al., 2014). Furthermore, knocking down *Ptbp1* in astrocytes can induce the generation of new dopamine-functional neurons in Parkinson’s disease mouse models, rebuild damaged neural circuits, restore dopamine levels in the striatum, and effectively alleviate motor impairments associated with Parkinson’s syndrome (Qian et al., 2020; Zhou et al., 2020). Conversely, some studies using lineage tracing methods have systematically demonstrated that overexpressing *NeuroD1* or knocking down *Ptbp1* does not result in the *in vivo* conversion of astrocytes into neurons (Wang et al., 2021).

The debate over astrocyte-to-neuron conversion continues, and this “contention of a hundred schools of thought” is beneficial as it promotes the dialectical advancement of science. We hope that more rigorous evidence in the future will demonstrate the *in situ* conversion potential of astrocytes into neurons, which could bring new hope for the treatment of neurodegenerative diseases.

### Mesenchymal stromal cell (MSC)-based therapy

MSCs have garnered attention because of their low immunogenicity, rapid proliferation rate, and wide range of sources. They can be isolated from a variety of tissues, including bone marrow, umbilical cord blood, adipose tissue, placenta, amniotic fluid, peripheral blood, and olfactory mucosa (Dominici et al., 2006). MSCs possess the potential to suppress immune cell dysfunction, encourage neurocompensatory growth, mitigate apoptosis, invigorate mitochondrial functions, and enhance cell survival rates, offering hope for the treatment of HD (Connor, 2018). A 2023 meta-analysis, which collated data from 15 studies involving 346 rodent subjects, demonstrated that MSC transplantation increased striatal volume, and notably, implantation before motor dysfunction onset was effective in obstructing HD-related motor deficits. Bone marrow-derived MSCs in particular displayed superior efficacy in motor coordination enhancement. However, significant improvements in cognitive function were largely absent after MSC implantation (Liang et al., 2023) (Table 5).

Research by Snyder et al. indicated that transplantation of human MSCs into the striatumof HD mice can stimulate the proliferation and neuronal differentiation of native NSCs (Snyder et al., 2010). Numerous studies have enhanced the therapeutic effects of MSCs through genetic modification or pre-treatment to improve their functionality. For instance, Dey et al. demonstrated that transplantation of MSCs overexpressing BDNF or nerve growth factor in YAC 128 model mice reduced neuronal loss in the striatum and ameliorated behavioral abnormalities (Dey et al., 2010). Similarly, Sadan et al. reported that transplanting neurotrophic factor (NTF)-overexpressing MSCs not only enhanced motor function in HD animal models but also extended lifespan of HD mice (Sadan et al., 2012a, 2012b). These findings suggest that neurotrophic factor-rich MSCs could provide a beneficial microenvironment for the striatum, thereby decelerating neuron degeneration.

Further studies, such as those by Linares et al., revealed that MSCs pre-treated with the mood stabilizers lithium and valproate augmented therapeutic efficacy in HD mice by enhancing motor abilities, decreasing striatal neuronal damage, and diminishing abnormal HTT aggregation (Linares et al., 2016). Moreover, Lin et al. revealed that human MSCs significantly alleviated motor dysfunction in HD mouse models by promoting neurogenesis, providing neurotrophic support, and exerting antiapoptotic effects, demonstrating their neuroprotective and restorative potential (Lin et al., 2011).

A recent innovative therapeutic avenue exploring the use of MSC-derived exosomes has shown promise. Exosomes are minuscule extracellular vesicles (EVs) released by cells into the extracellular environment that are capable of long-distance signalling and facilitating cell communication. EVs are packed with bioactive molecules such as proteins, lipids, mRNAs, and miRNAs that mirror the attributes of their parent cells and can modulate the behaviors and functions of adjacent cells. MSC-derived exosomes offer clear advantages in clinical use due to their small size, low immunogenicity, low tumorigenicity, and easier storage and transportation than whole cells (Ghasemi et al., 2023; Gorabi et al., 2019). These exosomes potentially contain molecules that facilitate neuronal cell survival, anti-inflammatory responses and cellular repair (Giampà et al., 2019). Further investigation into the functionality of MSCs could illuminate new cell therapy-free HD treatment approaches.

On the basis of their inherent nanotherapeutic properties, MSC-derived exosomes provide novel therapeutic tools for diverse neurodegenerative disorders. Advances in exosome content engineering to increase specificity and effectiveness may lead to the development of safer, more potent HD treatments. Continued exploration of this research area is likely to further reveal the therapeutic potential of MSC-derived exosomes, opening up new avenues for clinical treatment.

### Clinical trials based on cell therapy

Reports on cell therapy products for HD are somewhat rare. In this stimulating field, Cellavita has developed a noteworthy cell therapy product called NestaCell. This innovative treatment is derived from human immature dental pulp stem cells (hIDPSCs). Preliminary preclinical trials suggest that the administration of hIDPSCs can restore the expression of BDNF, DARPP32, and D2R, promoting neuroprotection and neurogenesis in an HD rat model (Wenceslau et al., 2022). The main clinical trials in Nestacell are shown in Table 4. The initial clinical trial, “SAVE-DH” (NCT02728115), was launched in Brazil by the company in 2017 and involved a cohort of six HD patients. Each patient received three intravenous doses of either 1 million cells per kg or 2 million cells per kg spanning a 4-month period. The trial monitored safety through the incidence, severity, and causal relationship of adverse events, measured efficacy using the UHDRS, CD4+ and CD8+ proliferation, and tested for inflammatory markers (cytokines). The subsequent phase II study, “ADORE-DH” (NCT03252535), focused on evaluating the dose-response impact of the investigational drug Cellavita HD on HD patients. The study utilized a 2:2:1 randomization ratio to administer either two doses or a placebo via three monthly intravenous injections over three cycles. Building upon these findings, the company launched an expanded phase II/III clinical trial, “ADORE-EXT” (NCT04219241), in February 2020 to assess the safety and efficacy of the maximum dosage (2 million cells/kg) of NestaCell treatment. This involved four treatment cycles, each comprising three intravenous injections. The cumulative findings from both the phase I (SAVE) and phase II (ADORE) trials showed that NestaCell significantly enhanced both motor scores and functional capacities in comparison to a placebo, indicating a clinically significant advantage. Coupled with a promising safety profile and minimal drug-related adverse events, NestaCell is a safe, well-tolerated treatment for HD patients within the tested dosage ranges (Joao et al., 2021). Looking forward, Azidus Brazil is set to initiate the phase III clinical trial “STAR (NCT06097780)” for NestaCell in June 2024. This will be a multicentre, double-blind, placebo-controlled, prospective randomized trial, aiming to recruit 120 participants and administer a higher dosage than those used in previous trials. The principal objective is to evaluate the safety and efficacy of this aggressive therapy, with the trial expected to conclude in 2026 (Kim et al., 2021).

### The challenges of cell therapy

Cell therapy represents a ground-breaking and auspicious treatment modality for HD. The transplantation of healthy cells into the brains of HD patients holds promise for replacing lost neurons and regulating the local milieu to bolster endogenous repair mechanisms. Nevertheless, cell therapy encounters various obstacles. First, therapeutic cell quantities are limited, and the challenge of sourcing cell lines compatible with good manufacturing practice (GMP) significantly hinders treatment feasibility. In numerous cases, the absence of long-term cell storage complicates quality control and coordination with surgical schedules. Additionally, the absence of standardized surgical protocols, appropriate administration techniques, and apprehensions regarding the safety and efficacy of postadministration cell products compound this issue. Moreover, the directed differentiation of transplanted cells and their seamless integration into the neural networks of the host brain continue to pose significant hurdles. Furthermore, the potential for tumorigenesis remains a concern owing to the incomplete differentiation of stem cells. Another pivotal consideration is the possible immune response triggered by the transplanted cells. Immune rejection or adverse immune reactions may undermine the effectiveness and safety of cell therapy interventions (D’Egidio et al., 2024; Ding et al., 2013; Sakthiswary and Raymond, 2012; Sivandzade and Cucullo, 2021; Yamanaka, 2020) (Table 3). To address these issues encountered in cell therapy for HD, the following strategies should be considered:

#### Optimization of the cell products

The optimal cell product depends on whether the therapeutic strategy is neuroprotective or involves neuroregeneration and/or circuit restoration. This involves enhancing the differentiation process to guarantee that the cellular products can mature into cell populations with the necessary characteristics and optimizing the GMP standardization protocol to ensure the batch stability of cell products and ultimately obtain high-quality cell products that pass various quality control standards (Food and Drug Administration, 2004, 2016). There could be safety issues when using cell products for human applications; for example, incorrectly modified cells might result in nonabsorption or tumor formation. In response to this challenge, compliance with GMP during the manufacturing and scaling-up processes is crucial. This involves translating basic research procedures into a GMP quality system, implementing quality control and assurance, and avoiding/minimizing reagents of animal origin. The manufacturing process must also define the quality attributes of the final product, such as cell number, viability, dose, phenotype, karyotype, copy number variation, and genomic stability (Ma et al., 2023).

#### Engineering cells

Immune rejection or adverse immune reactions can compromise the efficacy and safety of cell therapy interventions. Strategies to mitigate these immune responses, such as immunosuppressive regimens or engineered cells to evade immune detection (Anand et al., 2023; Morizane et al., 2017), require careful optimization to balance effectiveness with potential side effects. For instance, administering immunosuppressive drugs to patients, or eliminating key genes responsible for immune rejection through gene knockout techniques, can be achieved in iPSCs or hESCs. Additionally, gene editing strategies aimed at enhancing the safety and effectiveness of cell therapy must consider several factors, such as viral residue, off-target effects of gene editing, and the stability of gene expression. Optimizing experimental processes to completely eliminate viral particle residues, improving gene editing tools to reduce off-target effects, or incorporating conditional promoter elements to control the timing of gene expression can enhance the stability of gene expression.

#### Optimized delivery systems

The majority of clinical trials for cell therapy have employed simple needle/catheter devices. Several issues are associated with their use, including high donor cell mortality, undesirable outcomes due to cell reflux, and significant cell sedimentation. Although a stepwise design at the catheter tip has not been utilized for cell transplantation evaluation, it may have potential benefits (Chan et al., 2021; Krauze et al., 2005). During cell transplantation, uneven drug deposition and reflux are exacerbated by cell sedimentation. This effect may be partially mitigated by suspending the cells in a gel rather than a liquid solution, although this might increase the complexity of regulatory oversight (Malloy et al., 2017; Torres et al., 2015). Moreover, administration complexity, including needle tip diameter and delivery speed, can significantly affect cell differentiation (Kondziolka et al., 2011). Optimizing strategies for cell distribution, such as adding side holes to the delivery catheter, can improve process efficiency (Silvestrini et al., 2015).

#### Improving graft survival and integration

The survival and integration of grafts in host brain tissue require overcoming various obstacles. Beyond the previously mentioned issue of immune rejection, advancements in surgical techniques to reduce cell mortality, the use of neuroprotective agents, and the development of methods to enhance neural plasticity and circuit integration can significantly improve graft survival and integration efficiency (Cisbani et al., 2014; Harrower et al., 2006; Jgamadze et al., 2012; Winkler et al., 2017). Multiple studies have observed that transplanted neural stem cells or embryonic neurons form new synaptic connections with host neurons and successfully integrate into neural circuits in both rodent and primate brains (Falkner et al., 2016; Wei et al., 2016). For instance, Park et al. reported that co-transplanting regulatory T cells (Treg cells) with neurons alleviated damage during the transplantation process. Treg cells exerted neuroprotective effects by locally reducing inflammation, thereby increasing the survival rate of transplanted cells and promoting recovery in Parkinson’s disease (Park et al., 2023; Tang, 2023). This study introduces new avenues for cell therapy, and future exploration of additional methods could further enhance graft survival and integration.

#### Immunosuppression

Graft rejection poses a significant challenge in cell therapy. The recipient’s immune system may perceive the transplanted cells as foreign and mount a defensive response, resulting in graft rejection. Immunosuppressants are employed to dampen the immune response and prevent graft rejection (Chen and Palmer, 2008; Rong et al., 2014). Another approach to mitigate immune rejection involves transplanting cells with low immunogenicity, primarily through gene editing technologies to reduce the immunogenicity of hESCs and human iPSCs, a process that requires careful optimization for safety. Additionally, immune rejection can be minimized through autologous cell transplantation.

#### Generation and application of large HD animal models

A critical aspect of preclinical safety and efficacy studies involves the inclusion of large animal models. These models offer significant advantages over rodents due to their larger brain size and closer anatomical and functional similarity to the human brain. Utilizing large animal brains that resemble the human brain for cell therapy allows for more accurate predictions regarding the number and survival duration of transplanted cells, improvements in neural circuit connections, and a more objective assessment of clinically relevant adverse reactions following cell injection (Lunney et al., 2021). Furthermore, these models provide a platform for screening clinically applicable delivery systems and treatment strategies. Several studies have already employed non-human primate and porcine models of neurodegenerative diseases including HD to evaluate treatment strategies (Yan et al., 2023; Yang et al., 2019). The advancement of additional large animal models for HD is anticipated to pave the way for future therapeutic breakthroughs in HD treatment.

## Conclusion

With the advent of 3D culture techniques, researchers have developed methods to engineer tissue constructs mimicking the human striatum, known as human striatal organoids (hStrOs), from human iPSCs. These hStrOs manifest the potential for cellular diversity and enhanced directed differentiation towards MSNs (Chen et al., 2022; Miura et al., 2020). Organoids generated from human iPSCs approximate aspects of human embryonic development to a certain degree, potentially improving compatibility with the host brain and suitability for cell transplantation therapies. Moreover, the dynamic 3D culture process can provide ecological niche signals at appropriate stages to induce the formation of specific cell types with more complete and mature functionality (Li and Izpisua Belmonte, 2019). Despite these advancements, no studies have confirmed whether hStrOs can be used as donor grafts for the treatment of HD. Future work should focus on confirming the safety, efficacy, and therapeutic potential of these organoids both in animal HD models and patients.

As mentioned above, current research on the use of MSCs for treating CNS diseases suggests that their therapeutic effects are largely attributed to their secretory and supportive properties. As a natural constituent of the cellular niche, MSCs can influence the environment through immunomodulation and the secretion of trophic factors. They can home to damaged regions of the brain, eliciting neuroprotective and angiogenic effects. Additionally, MSCs can promote the regeneration of cells in damaged cerebral cortex, and their secretomes can have a positive impact on neuronal recovery following traumatic brain injury (Anbari et al., 2014; Chuang et al., 2012; Mathot et al., 2020; Obtulowicz et al., 2016). Therefore, when considering cell transplantation, the co-transplantation of MSCs could be explored, leveraging their immunomodulatory and trophic effects to mitigate the mechanical damage caused during the transplantation process, minimize immune rejection, and enhance the survival and targeted differentiation of functional cells post-transplantation.

Microglia are self-renewing immune cells in the CNS that play crucial roles in brain development, homeostatic regulation, and disease pathogenesis (Bohlen et al., 2019). Under normal physiological conditions, they participate in synaptic pruning through phagocytosis, aiding in the formation of accurate neural circuits, while also secreting BDNF to promote the formation of dendritic spines (Paolicelli et al., 2011; Parkhurst et al., 2013). However, in neurodegenerative diseases, genetic mutations can cause microglia to shift from defenders to neurotoxic aggressors, triggering excessive proinflammatory responses and neurotoxicity that accelerate the progression of neurodegenerative disorders (Hickman et al., 2018). Consequently, eradicating these dysfunctional microglia might represent a novel therapeutic approach for treating neurodegeneration. Studies suggest that repopulating microglia can exert neuroprotective effects and support neuronal differentiation from NSCs by restoring BDNF signalling pathways (Wang et al., 2020, 2023). Thus, repressing microglia carrying mutant genes during cell transplantation could facilitate the engraftment and targeted differentiation of transplanted cells and might even contribute to the formation of new neurons and synapses. Nonetheless, the potential of these innovative strategies requires further substantiation through research.

**Figure 4.**
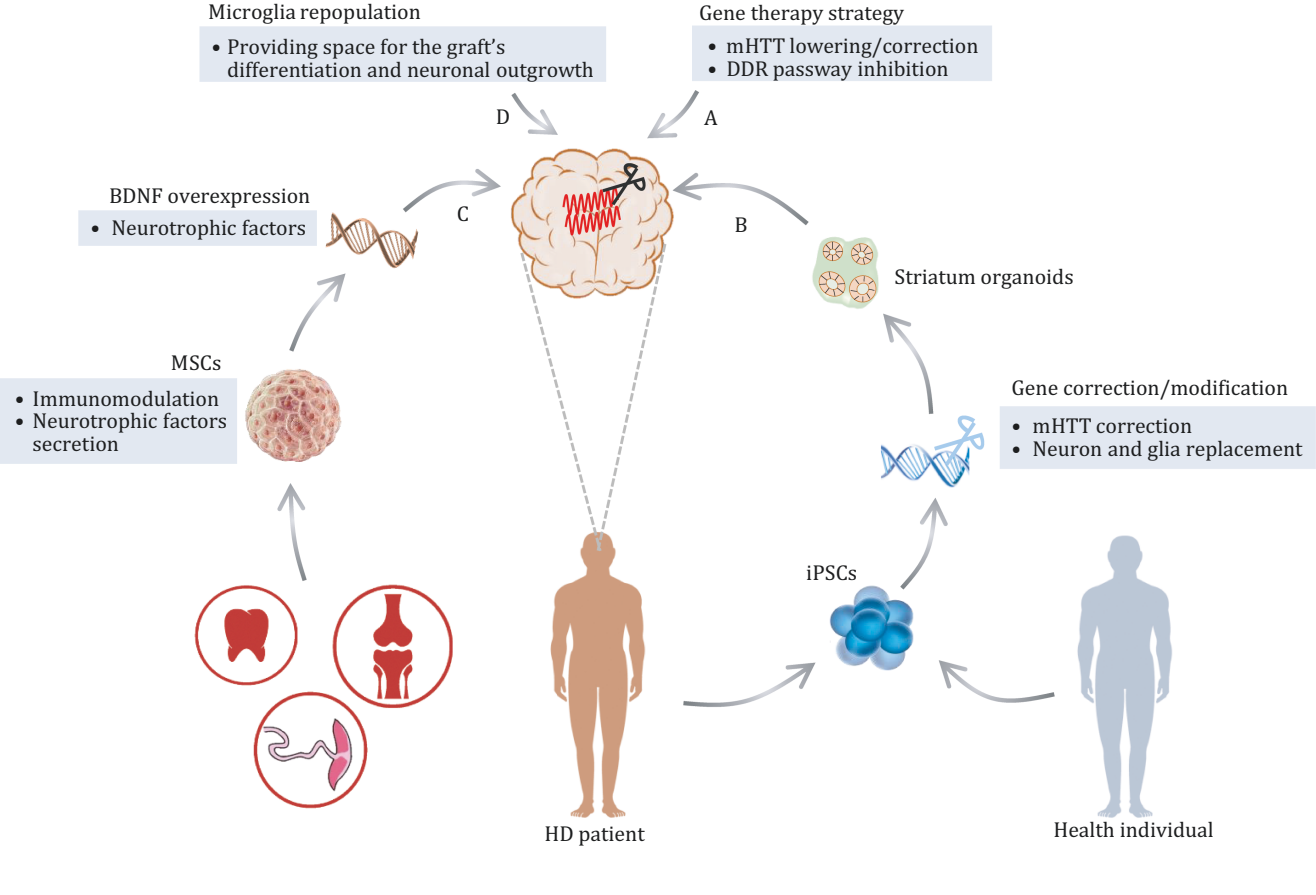
Potential future applications of therapeutic strategies for Huntington’s disease. (A) Gene therapy strategies aim to directly target patients for the reduction or correction of mHTT or inhibition of the DDR pathway. (B) Combining cell therapy with gene therapy involves replacing damaged neurons and glial cells with striatal organoids derived from iPSCs obtained from healthy individuals or gene-corrected HD patients. (C) To minimize needle-mediated inflammatory responses during cell transplantation, MSCs are co-transplanted to modulate the immune microenvironment. Additionally, the overexpression of BDNF in MSCs can contribute to therapeutic effect. (D) The repopulation of microglia provides the transplanted cells with the space needed for differentiation and neuronal outgrowth.

Considering the multifaceted nature of HD, a combination therapy targeting different cellular, epigenetic, and genetic factors could improve the mitigation of HD symptoms. For example, hStrOs derived from hiPSCs, which are similar to embryonic development (Li and Izpisua Belmonte, 2019; Miura et al., 2020), facilitate their targeted differentiation and effective integration with the host brain’s neural network post-transplantation. Co-transplantation of hStrOs with human umbilical cord derived MSCs (hUC-MSCs) leverages the immunomodulatory and neurotrophic factor secretion capabilities of hUC-MSCs (Pischiutta et al., 2022), mitigating mechanical damage and immune rejection during the transplantation process. This approach enhances the survival rate and targeted differentiation of transplanted cells. Additionally, the repopulation of microglia can provide a supportive microenvironment for the grafted cells, promoting effective integration between the graft and the host (Fig. 4). Combined therapeutic approaches may offer synergistic benefits beyond those of single therapies. However, recent pre-clinical studies have predominantly investigated this combination approach to a limited extent.

The next crucial step is designing clinical trials to assess the safety and efficacy of innovative strategies (e.g., epigenetic, genetic, and stem cell-based approaches) that have shown great potential for modifying the course of HD. The promising preclinical results underscore their potential candidacy for future clinical trials. By refining these strategies, the quest for a treatment for this devastating disorder may soon be realized.
