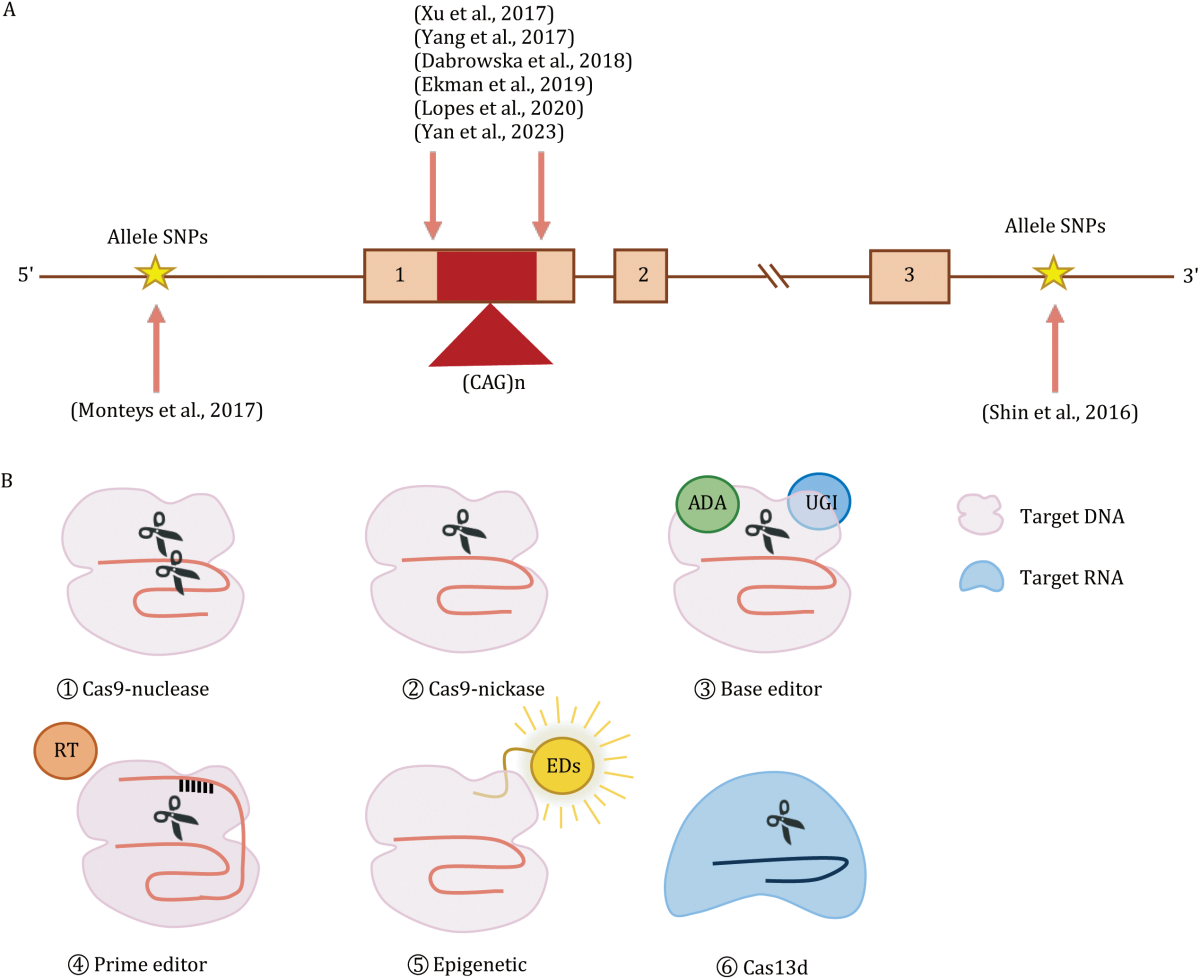# Correction to: Advances in gene and cellular therapeutic approaches for Huntington’s disease

**DOI:** 10.1093/procel/pwaf005

**Published:** 2025-02-12

This is a correction to: Xuejiao Piao, Dan Li, Hui Liu, Qing Guo, Yang Yu, Advances in gene and cellular therapeutic approaches for Huntington’s disease, *Protein & Cell*, 2024; https://doi.org/10.1093/procel/pwae042

In the version originally published, in Figure 3 the key indicating symbols for target RNA and target DNA was reversed. This error has been corrected in Figure 3 within article.